# Hierarchical structure formation by crystal growth-front instabilities during ice templating

**DOI:** 10.1073/pnas.2210242120

**Published:** 2023-05-31

**Authors:** Kaiyang Yin, Kaihua Ji, Louise Strutzenberg Littles, Rohit Trivedi, Alain Karma, Ulrike G. K. Wegst

**Affiliations:** ^a^Thayer School of Engineering, Dartmouth College, Hanover, NH 03755; ^b^Department of Physics, Northeastern University, Boston, MA 02115; ^c^Department of Microsystems Engineering, University of Freiburg, 79110 Freiburg, Germany; ^d^Center for Interdisciplinary Research on Complex Systems, Northeastern University, Boston, MA 02115; ^e^Materials Science and Metallurgy Branch, NASA Marshall Space Flight Center, Huntsville, AL 35812; ^f^Department of Materials Science and Engineering, Iowa State University, Ames, IA 50011

**Keywords:** freeze casting, faceted crystals, anisotropic growth, phase-field modeling

## Abstract

Directional solidification of aqueous solutions and slurries in a temperature gradient is widely used to produce cellular materials through a phase separation of solutes or suspended particles between growing ice lamellae. While this process has analogies to the directional solidification of metallurgical alloys, it forms very different hierarchical structures. The resulting honeycomb-like porosity of freeze-cast materials consists of regularly spaced, lamellar cell walls which frequently exhibit unilateral surface features of morphological complexity reminiscent of living forms, all of which are unknown in metallurgical structures. While the strong anisotropy of ice-crystal growth has been hypothesized to play a role in shaping those structures, the mechanism by which they form has remained elusive. By directionally freezing binary water mixtures containing small solutes obeying Fickian diffusion, and phase-field modeling of those experiments, we reveal how those structures form. We show that the flat side of lamellae forms because of slow faceted ice-crystal growth along the c-axis, while weakly anisotropic fast growth in other directions, including the basal plane, is responsible for the unilateral features. Diffusion-controlled morphological primary instabilities on the solid-liquid interface form a cellular structure on the atomically rough side of the lamellae, which template regularly spaced “ridges” while secondary instabilities of this structure are responsible for the more complex features. Collating the results, we obtain a scaling law for the lamellar spacing,  $\lambda ∼{\left(VG\right)}^{-1/2}$ , where V and G are the local growth rate and temperature gradient, respectively.

## Significance

Ice templating is the mechanism by which cellular materials with hierarchical architectures are formed when aqueous solutions or slurries are directionally solidified or freeze cast. Characteristic for freeze-cast solids are lamellar cell walls with unilateral surface structures such as regularly spaced “ridges” and other complex subfeatures that do not reflect the underlying hexagonal symmetry of ice. How these unilateral structures form has remained a mystery. Combining experiments and computational modeling, we reveal that, by localizing diffusion-limited morphological instabilities to the atomically rough side of ice lamellae, anisotropic ice-crystal growth templates hierarchical architectures with complex subfeatures. Those results lay the theoretical foundation for understanding the formation of hierarchical architectures and structural subfeatures produced by directionally solidifying aqueous mixtures.

**Fig. 1.**
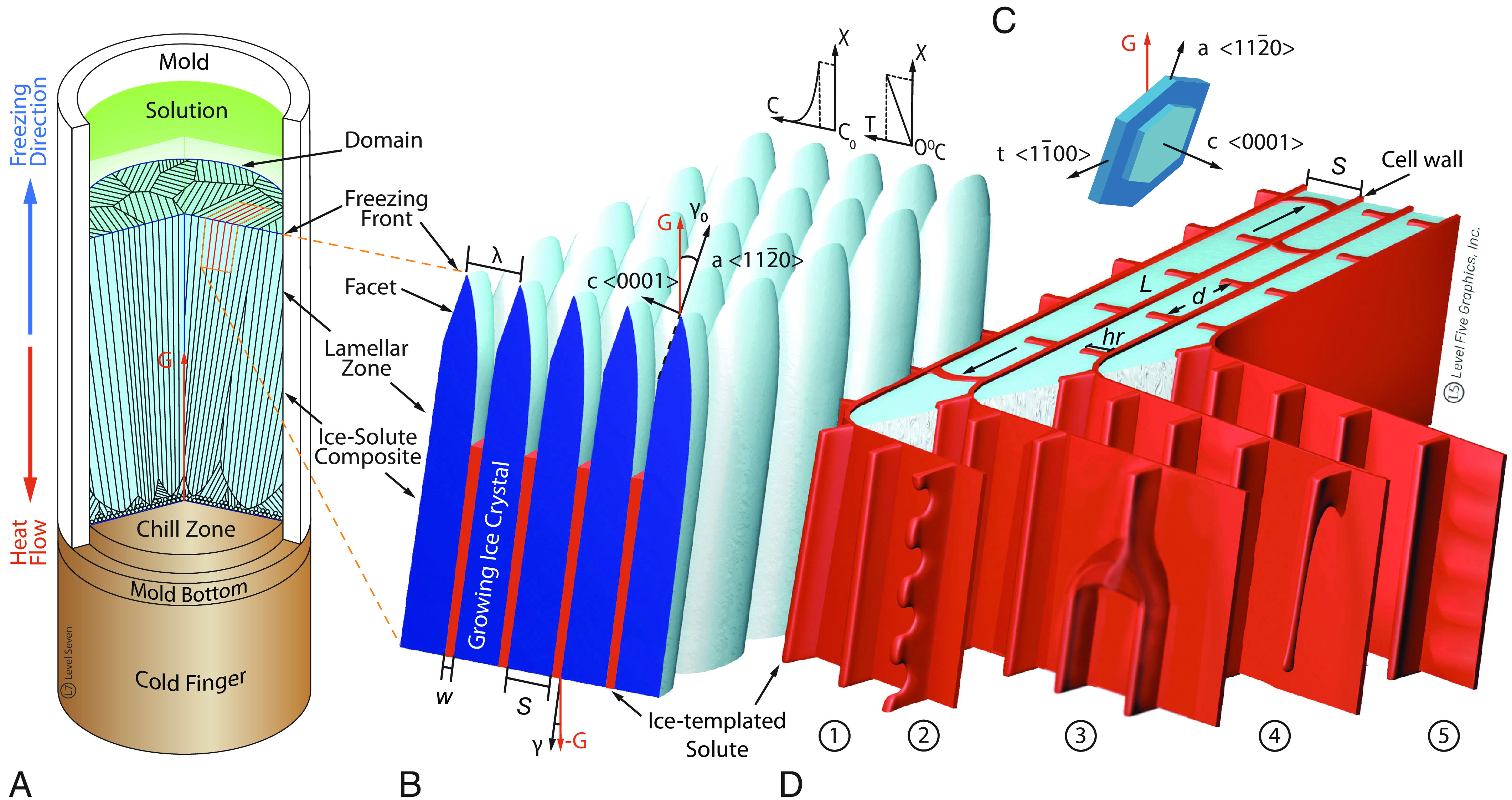
Schematic of the freeze casting process. (*A*) A mold, sealed with a copper bottom plate, is filled with an aqueous solution and placed on a cold finger, with which a controlled cooling rate is applied. Ice-crystals nucleate typically on the mold bottom and, due to rapid cooling, first form a textureless chill zone of fine, equiaxed grains, then grow competitively in the general direction of maximum heat flow imposed by the thermal gradient, *G* (8, 14). Those grains, whose fastest crystal growth direction is most closely aligned with the gradient, win; their size and orientation define the size of the domains of uniform lamellar orientation. Each of the winning grains grows with an initially approximately planar freezing front (15–17). (*B*) When ice-crystals nucleate and the freezing front progresses with a velocity, V , a phase separation into ice-crystals and a solute occurs. The solute is partitioned into the liquid phase so that the freezing front pushes ahead of it an exponentially decaying concentration profile, *C* > *C*_0_, where *C*_0_ is the initial solute concentration, with solutal diffusion length, $l_{D}=D/V$ , where *D* is the diffusion coefficient in the liquid phase (Fig. 1*B*, *Inset*) (18). (*C*) Hexagonal ice-crystal, *I*_h_. (*D*) In addition to the pore morphology, the growing ice-crystals template predominantly unilateral features on the cell walls: (1) smooth “ridges” that sometimes (2) partially detach, triangular, “shark-teeth”-like undulating tips; (3) “jellyfish”-like caps, where two “ridges” merge and continue as one; (4) “jellyfish-tentacle”-like protrusions, which may be partially or completely detached along their length, and form part of a “jellyfish-array” or ridge; and (5) wavy or wrinkled cell walls.

Freeze casting is a directional solidification technique that exploits the phase separation of water-based solutions or slurries into pure ice-crystals and a solid solute-rich amorphous phase (Fig. 1*A*). During freezing, dissolved solutes and suspended particles are upconcentrated and templated between the growing ice-crystals until glass transition is reached, and vitrification occurs (Fig. 1*B*). Removing the ice phase by sublimation after solidification reveals the complex hierarchical architectures of the “ice-templated” cellular materials, which is the basis of their attractive property profiles (1–3). Recent systematic studies highlight that not only pore size and morphology, but also predominantly unilateral features, i.e., features forming only on one side of the cell walls (Fig. 1*D*), determine the mechanical and physical properties of ice-templated materials (4–6) rendering them attractive for applications ranging from those in biomedicine (4, 7–10) to those in energy generation and storage (11–13).

Exactly how the complex ice-templated structures form is not fundamentally understood beyond the very early stages of morphological development (19–22). As the ice-liquid interface grows with an initially planar morphology, it rejects solute that builds up ahead of the interface. When the ratio V/G of the interface velocity, V , and temperature gradient, G , parallel to the heat flow direction exceeds some threshold, the liquid ahead of the interface becomes thermodynamically unstable [constitutionally supercooled (20)]. This provides the driving force for phase separation which in turn triggers a morphological instability of the solid-liquid interface first analyzed by Mullins and Sekerka (19) in the metallurgical context of alloy solidification. Small amplitude wavy sinusoidal perturbations of the interface become amplified at a preferred wavelength, $\lambda_{0}$ , that reflects the competition between the destabilizing effect of solute diffusion in the liquid and the restabilizing effects of capillarity and the temperature gradient at small and large wavelengths, respectively.

While the Mullins-Sekerka linear stability analysis has been extended to freeze casting (21–23), it has remained a complete mystery how the resulting lamellar array of growing ice-crystals templates complex hierarchical structures with unilateral features (Fig. 1*D*). Recent computational modeling studies of ice templating have been limited to two dimensions (2D) and have, so far, not reproduced those features (24, 25). Comparatively much better understood is microstructural pattern formation during directional solidification of metallic alloys, which form traditional cellular and dendritic array structures after initial morphological instabilities (17, 26–29). In those simpler alloy systems, the structure of the solid-liquid interface is atomically rough for all orientations, leading to a weak surface tension anisotropy and gently curved crystallization fronts that have been modeled quantitatively (30–34). Moreover, both the solvent and solutes are of atomic size with well-characterized alloy thermodynamic properties.

In contrast, the ice-liquid interface is faceted (atomically smooth) perpendicular to the c-axis and atomically rough in the other directions contained in the basal plane (15, 35). As a result, similar to snowflake growth from a super-saturated water vapor, ice-crystal growth from the melt is strongly anisotropic. Growth is much faster along the twelve hexagonal directions than along the c-axis (Fig. 1*C*). Additionally, the chemistry and transport properties of the solutions or slurries used for freeze casting applications are more complex than liquid mixtures of atomic elements. The solutes in polymeric systems are frequently large network-forming molecules or a mixture of large and small molecules, with concentration-dependent diffusion coefficients. Ceramic or metal slurries are typically composed of particles of different sizes and at least one, more frequently two large polymers acting as dispersant and binder. During the directional solidification of such solutions and slurries, a mixed-phase region (mushy layer or zone) forms, in which an increasingly viscous solute or solute-bound particle phase alternates with the ice dendrites (22, 36, 37). Morphological instabilities and complex ice-crystal microstructures due to constitutional supercooling can form also in this mushy layer, which is defined by a concentration-dependent diffusivity, a nonlinear freezing temperature curve, and transport phenomena specific to colloidal mushy layers (21–23, 36).

While anisotropic ice-crystal growth, which tends to select the fastest $\langle 11\bar{2}0\rangle$ growth directions to parallel the temperature gradient, can qualitatively account for the predominantly lamellar morphology of the ice-crystals, it falls short of explaining the formation of substructures that unilaterally decorate the cell walls. Similarly, anisotropic growth falls short of explaining the mechanisms controlling the pore structure and lamellar spacing, λ , on a larger scale (Fig. 1*B*), with the lamellar spacing known to be unrelated to and much larger than $\lambda_{0}$ in the alloy solidification context (38). Additionally, it remains unknown to what degree the anisotropic solidification properties of ice and solution properties contribute to hierarchical structure formation in comparison to other effects, such as inter-lamellar fluid and shear flows, induced by both the 9% volume expansion during the phase transition from water to ice and mold contraction, which are known to align molecules and anisotropic particles (39–41).

Herein, we present experimental and computational modeling results that focus on the fundamental question of the formation of unilateral substructures on the cell walls. We demonstrate experimentally that freeze casting of solutions with simple chemistries, in particular small solute molecules, which obey simple Fickian diffusion in the liquid, produces hierarchical architectures with elaborate substructures (Fig. 1*D*) similar to those obtained with more complex chemistry. We further use fully three-dimensional (3D) computational modeling with a new phase-field (PF) formulation of ice templating that incorporates quantitatively the anisotropic properties of ice-crystal growth in simple binary solutions. The simulations reveal how templating those structures results from interfacial pattern formation mechanisms directly linked to the anisotropic growth properties of ice in simple binary solutions.

## Hierarchical Templated Structures from Simple Solution Chemistry

The presence or absence of the various cell wall features in freeze cast materials depends generally on solution and slurry composition and processing conditions (2–5). In the most frequently studied particulate systems (e.g., freeze-cast ceramics and metals), the highly regular features observed in purely polymeric systems tend to be obscured by additional phenomena such as sedimentation and particle-freezing-front interactions, which are particle size dependent and affect particle packing both within the cell wall and on the cell wall surface (2, 39, 42, 43). Regular unilateral surface features, rather than a unilateral surface roughness, are observed only in material systems in which the particle radius, R , is considerably smaller than the short pore dimension, S=(1-f)λ (Fig. 1*D*), where f is the solid fraction (44). Furthermore, since particulate systems are typically quaternary (composed of a solvent, particles, a dispersant, and a binder), the individual effect of the different components on structure formation cannot easily be distinguished. Finally, empirical correlations between ice-templated structure and processing parameters in particulate systems (e.g., between applied cooling rate and pore morphology) are, with few exceptions (e.g., refs. 39 and 43), obtained after the additional processing step of sintering (45); the latter is problematic because structural parameters such as cell wall thickness and lamellar spacing are not only determined by the freezing rate but also the sintering conditions (43).

To avoid these various complications, we investigated two distinct chemistries that have well-characterized phase diagrams (*SI Appendix*, Fig. S1) and solute transport properties (*SI Appendix*, Fig. S2). Importantly, for both chemistries, R≪S , thereby enabling us to i) perform detailed observations of fine hierarchical features, and ii) identify the minimal ingredients of solution chemistry contributing to their formation. The first is the ternary chitosan-acetic-acid-water system, where chitosan is a large polymer with R ∼ 300 nm and acetic acid is a small molecular size additive. The second is the much simpler binary water-sugar system (water-sucrose and water-trehalose) with a considerably smaller R of only a few nm. The diffusion constant, D , is two orders of magnitude smaller for chitosan than for the fast-diffusing sugars, consistent with the well-established Stokes-Einstein relation $D\approx k_{B}T/\left(6\pi R\eta \right)$ , where η is the viscosity of water (*SI Appendix*, Fig. S2). As a result, in the chitosan-acetic-acid-water system, diffusive transport of the large submicron polymeric particles is too slow at dilute solute concentration (small particle volume fraction) to keep up with the rate of ice-crystal growth. However, after an initial pile-up of the particles, transport becomes dramatically enhanced as the volume fraction approaches the close-packing limit due to particle-particle interactions. This enhancement is reflected in a strongly nonlinear relationship between effective diffusion constant and volume fraction (22, 36, 37). Taking into account this nonlinear relationship is therefore essential to quantitatively describe the growth of ice fronts enveloped by a dense layer of nearly close-packed particles. In contrast, in the water-sugar system, diffusion of the small solute particles is sufficiently fast at dilute concentration to avoid pile-up. Instead, as in metallurgical alloys forming cellular/dendritic structures (19, 20, 26, 38, 46), a Fickian diffusion boundary layer is formed ahead of the growing ice front, characterized by an exponential decrease of concentration with distance away from the front on a scale $l_{D}=D/V$ (Fig. 1*B*).

**Fig. 2.**
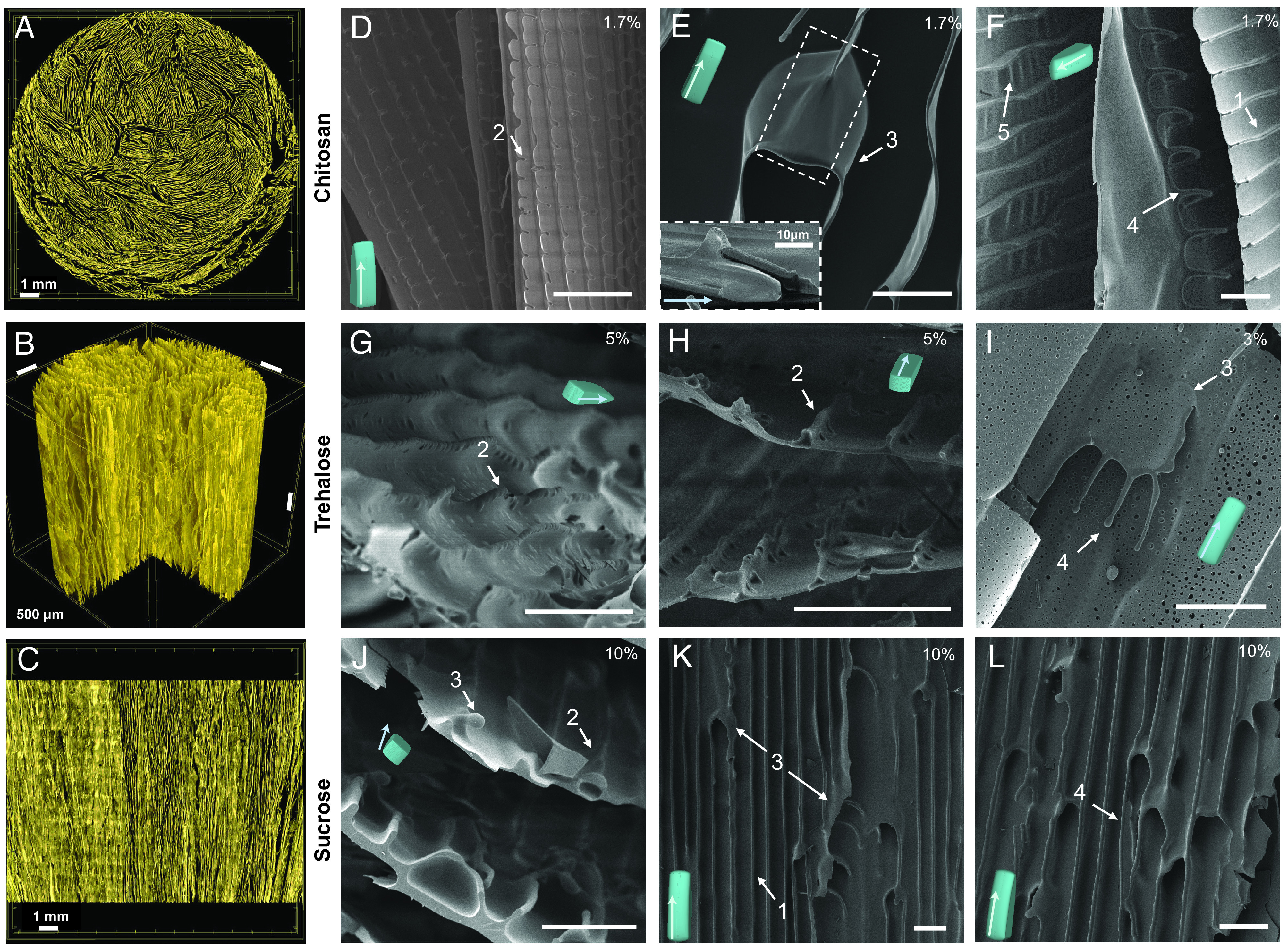
cell wall features observed in the binary model systems sucrose-water and trehalose-water (small molecules), in comparison to those observed in chitosan (large molecule). (*A*) Cross-section perpendicular to the freezing direction, (*B*) volume rendering, and (*C*) cross-section parallel to the freezing direction of a 3% w/v trehalose scaffold freeze-cast at an applied cooling rate of 1 °C/min, imaged by X-ray microtomography. (*D*–*L*) SEM micrographs of typical features observed on cell walls of (*D*–*F*) chitosan (freeze cast at 1 °C/min), (*G*–*I*) trehalose scaffolds, and (*J*–*L*) sucrose scaffolds (both freeze cast at 10 °C/min); the respective solute concentrations is marked in the top right corner of each micrograph: (1) smooth “ridges,” (2) “shark-teeth,” (3) “jellyfish-caps,” (4) “jellyfish-tentacles,” and (5) wavy or wrinkled cell walls. The scale bar is 50 µm in (*D*–*L*).

Experimental observations reported in Fig. 2 reveal that, remarkably, freeze-cast templated structures formed in the large-polymer chitosan and small-polymer sugar-in-water solutions exhibit the same scaffold architecture and unilateral cell wall surface features. A careful qualitative and quantitative structural characterization of the hierarchical scaffold architecture of a trehalose scaffold by X-ray tomography (Fig. 2 *A*–*C*), and of the cell wall surfaces by SEM of chitosan (Fig. 2 *D*–*F*), trehalose (Fig. 2 *G*–*I*), and sucrose (Fig. 2 *J*–*L*), shows that all exhibit five unique, typically unilateral microstructural features. These features are introduced and numbered in the schematic of Fig. 1*D*. Commonly observed are smooth “ridges” 1), and four additional features including the sometimes partially detached, triangular, “shark-teeth”-like undulating “ridges” 2), “jellyfish”-like caps, where two “ridges” merge and continue as one 3); “jellyfish-tentacle”-like protrusions 4) that may be partially or completely detached along their length and form part of a “jellyfish” array or “ridge”; and wavy or wrinkled cell walls 5); see schematic of Fig. 1*D* for comparison.

## Physical Model

The observation of the same scaffold architecture and unilateral features across these disparate aqueous material systems suggests that their formation is determined by the strongly anisotropic growth properties of ice; the strongly anisotropic growth properties of ice distinguish the water-sugar binary systems with fast-diffusing solutes from metallic alloy systems with weakly anisotropic solid-liquid interface properties (26). To test this hypothesis, we examine the directional solidification (Fig. 1) of the sucrose-water and trehalose-water systems, ignoring the second processing step in freeze casting, the removal of the ice phase from the ice–polymer composite by sublimation (lyophilization), which only consolidates the ice-templated structure by further drying (3, 47).

Directional solidification is controlled by a standard set of sharp-interface equations that consists of i) the diffusion equation in the liquid phase, $\partial_{t}c=D\nabla^{2}c$ , where c is the solute concentration, ii) the condition of mass conservation (Stefan condition) at the solid-liquid interface, $c_{l}V_{n}=-D\partial_{n}c{|}^{+}$ , where $c_{l}$ is the solute concentration on the liquid side of the interface, $V_{n}$ is the normal interface velocity, and $\partial_{n}c{|}^{+}$ is the gradient of concentration along a direction normal to the interface pointing towards the liquid side, and iii) a velocity-dependent form of the Gibbs-Thomson relation

[1] $$T_{I}=T_{M}-\left|m\right|c_{l}-\frac{T_{M}}{L}\sum_{i=1}^{2}\left[\gamma \left(n\right)+\gamma_{\theta_{i}\theta_{i}}\left(n\right)\right]K_{i}-\frac{V_{n}}{\mu_{k}\left(n\right)},$$

where $T_{I}$ is the local interface temperature, $T_{M}$ is the melting point of pure ice and L is the latent heat of fusion per unit volume, m is the liquidus slope assumed constant in the dilute solution limit of the water-sugar phase diagrams, $\gamma \left(n\right)$ is the excess free-energy of the solid-liquid interface, which is anisotropic due to its dependence on the direction normal to the interface n , $K_{i}$ and $\theta_{i}$ are the two principal interface curvatures and the two angles between and the principal directions and n , respectively, and $\mu_{k}\left(n\right)$ is the anisotropic atomic attachment kinetic coefficient.

The functional dependencies of $\gamma \left(n\right)$ and $\mu_{k}\left(n\right)$ on orientation are chosen to represent the known strongly anisotropic properties of the ice-water interface for hexagonal ice (Fig. 1*C*), which is atomically rough in the directions contained within the basal plane, including the six preferred $\langle 11\bar{2}0\rangle$ growth directions (a-axes) and the six $\langle 1\bar{1}00\rangle$ prism directions (t-axes), but atomically smooth (faceted) in planes perpendicular to the c-axis. Accordingly, when n is contained in the basal plane, $\gamma \left(n\right)$ is weakly anisotropic and interface kinetics is sufficiently fast for the kinetic undercooling $V_{n}/\mu_{k}\left(n\right)$ to be neglected; in this plane growth is in local equilibrium with the weak anisotropy of $\gamma \left(n\right)$ favoring growth along $\langle 11\bar{2}0\rangle$ similar to the weak anisotropy of $\gamma \left(n\right)$ selecting $\langle 100\rangle$ directions in metallic systems with cubic symmetry (30, 48, 49). In contrast, the ice-water interface is atomically smooth in planes perpendicular to the c-axis. As a result, growth in the $\langle 0001\rangle$ direction is much more sluggish and controlled by processes such as two-dimensional island nucleation or spiral growth around screw dislocations (50). These processes lead to a large kinetic undercooling on facets that is determined by a temperature-dependent form of $\mu_{k}$ (35). Accordingly, we choose a strongly anisotropic form of $\mu_{k}\left(n\right)$ that equals its experimentally measured value for slow growth along $\langle 0001\rangle$ , and increases rapidly with n away from $\langle 0001\rangle$ so that $V_{n}/\mu_{k}$ becomes negligibly small for growth in this plane. In addition, we use the standard frozen temperature approximation that neglects latent heat rejection and assumes a fixed temperature gradient, G , corresponding to equally spaced isotherms moving at a fixed V . Accordingly, the temperature field along the vertical *x* axis in the moving frame takes the form $T\left(x\right)=T_{0}+G\left(x-Vt\right)$ , where $T_{0}$ is the reference temperature at *x* = 0 and *t* = 0.

To solve the above 3D free-boundary problem of ice templating, we use the PF-method that has been widely used to model alloy solidification patterns in a metallurgical context (31–33). This method has the main advantage that it circumvents the notorious difficulties of front tracking by making the solid-liquid interface spatially diffuse. However, using this method to make quantitative predictions is more challenging. It generally requires mapping the PF-model equations and parameters to the original free-boundary problem in a thin-interface limit where the width of the spatially diffuse interface is smaller than the characteristic scale of the microstructure, in order to resolve the pattern formation process, but much larger than the physical atomic scale width of the crystal-melt interface for simulations to remain computationally tractable. While this difficulty has been overcome for the solidification of binary alloys with atomically rough solid-liquid interfaces in the limit of local thermodynamic equilibrium at the interface (51, 52), where the kinetic undercooling $V_{n}/\mu_{k}\left(n\right)$ in Eq. **1** is negligibly small, modeling ice-templating poses the additional challenge of modeling simultaneously this limit for growth in the basal plane and slow faceted growth with a large kinetic undercooling along the c-axis. To overcome this difficulty, we have extended in a non-trivial way the PF formulation of refs. 51 and 52 to recover the desired interface boundary condition (1) without and with kinetic undercooling for growth in the basal plane and along the c-axis, respectively. The approach is summarized in the *Materials and Methods* section and the equations of the model are listed in the *SI Appendix*. Technical details of the thin-interface asymptotic analysis mapping the PF-model to the free-boundary problem of ice templating and numerical tests of convergence as a function of interface thickness deserve a longer exposition and will be presented elsewhere.

As input for the PF-simulations, we experimentally determined *G* and V (*SI Appendix*, Table S1) for the sucrose system, and diffusion coefficients, *D*, for sucrose, trehalose, and chitosan (*SI Appendix*, Fig. S2). In addition, we use the Clausius-Clapeyron relation for dilute alloys to estimate the analytical values of the liquidus slope (53), which are compared to the experimentally measured phase diagrams of sucrose and chitosan in *SI Appendix*, Fig. S1. The ternary chitosan-acetic-acid-water system is treated as a pseudo-binary system with only acetic acid as the mobile solute. In addition, for all aqueous systems, the interface kinetic coefficient for growth along the c-axis is determined from experimental measurements of basal plane growth (35, 54, 55) and the excess interface free-energy is extracted from molecular dynamics simulations (56) (*SI Appendix*). The PF-model domain is a region from 50 μm above to 300 μm below the solidification front.

Although the glass transition occurs in deep grooves between ice-crystal structures several millimeters away from the solidification front, the complex templated structures are primarily shaped in a region near this front where the key morphological instabilities of the solid-liquid interface form. Thus, by focusing on the ice-crystal growth in a sufficiently large region of the solidification front in the PF-modeling (several hundred microns along the *z* axis), we can reasonably assume that the preliminary ice-templated structure is represented by the unfrozen portion of the simulation domain. In addition, since crystals and grains with a large misorientation tend to be eliminated by early growth competition, the surviving ice-crystals typically grow with a small misorientation angle γ_0_ ≤ 10° with respect to the applied thermal gradient (3, 44). We report here PF-simulation results for γ_0_ = 10° that reproduce the main structural characteristics of freeze-cast materials, including the overall honeycomb-like pore architecture and the rich variety of unilateral features on the polymer cell walls. We find that all cell wall surface features predominantly face towards the warm end of the mold, as observed (4, 44), when γ_0_ exceeds a small threshold value of a few degrees. Hence, results for γ_0_ = 10° are representative of most growing crystals with a small finite misorientation. Additional results over a complete range of γ_0_ values will be presented elsewhere.

## Cell Walls and Unilateral “Ridges”

**Fig. 3.**
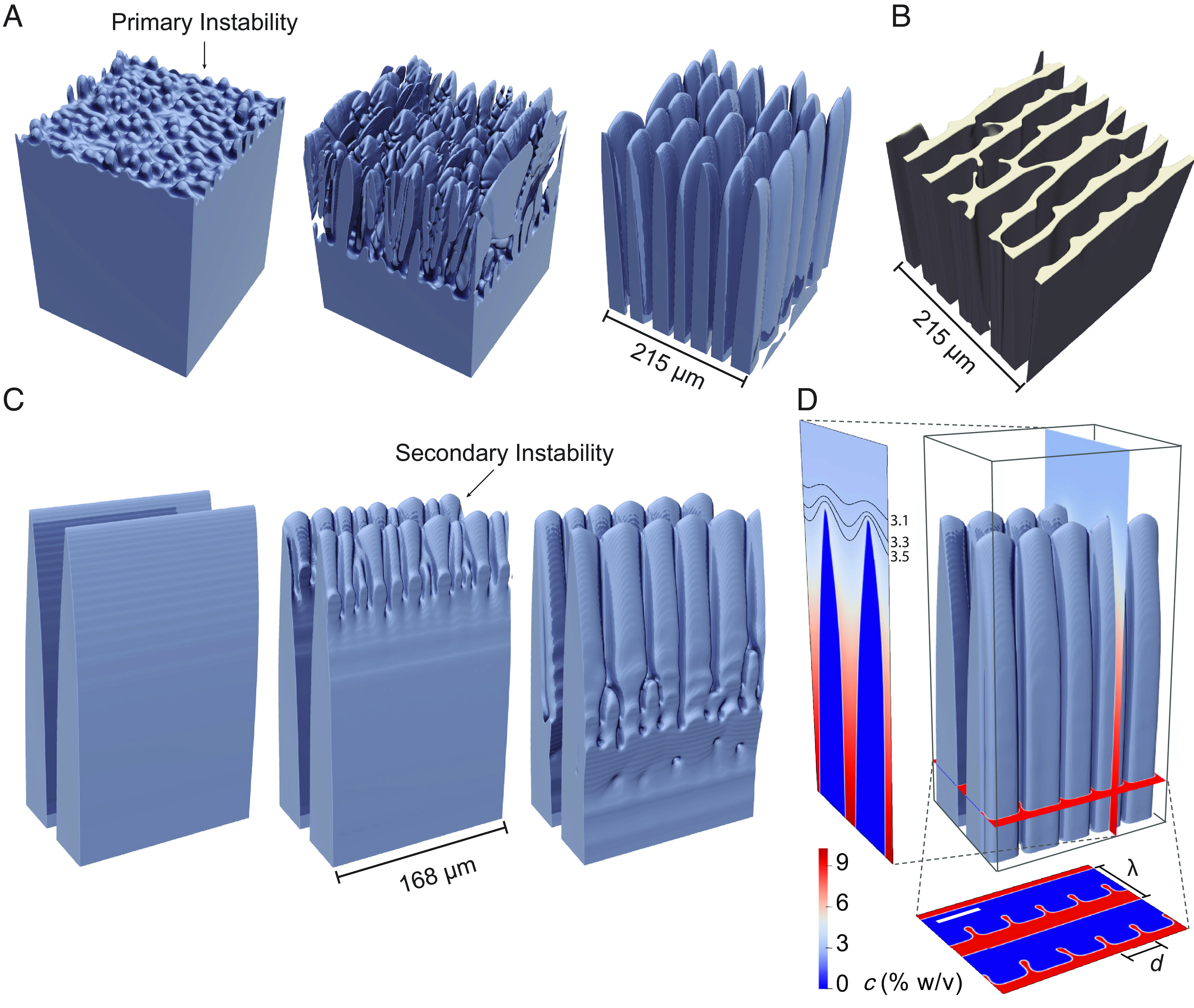
Formation of ice lamellae in PF-simulations during unidirectional freezing of an aqueous solution. (*A*) Structure formation captured at *t* = 18 s (*Left*), 22 s (*Center*), and 101 s (*Right*) in the 3D PF-simulation with a planar interface initially located at its steady-state position (*T = T_0_*). As the planar interface breaks down at the time, *t* = 18 s, the primary instabilities that are qualitatively similar to the classical Mullins-Sekerka instability of binary-alloy solidification can lead to protuberances with small wavelengths. These protuberances develop into a lamellar structure with a larger wavelength (primary spacing λ ). (*B*) Porous structure of the solute templated by ice-crystals in (*A*). (*C*) Structure formation captured at *t* = 0 s (*Left*), 9 s (*Center*), and 19 s (*Right*) in the 3D PF-simulation with extended 2D lamellae as the initial condition. The accompanying movie is included in *SI Appendix* (Movie S1). (*D*) Asymmetric ice lamellae and the cross-sections. The interface of the ice-crystal is shown in a dar k blue color. The cross-sections are shown by colormaps of solute concentration. The scale bar in the horizontal projection represents 35 μm. The black curves in the vertical projection represent iso-concentration lines with numbers on the side indicating the concentration in the unit of % w/v. In all PF-simulations, the growth conditions for freezing an aqueous 3% w/v trehalose solution are temperature gradient *G* = 12 K/cm and growth velocity *V* = 15 μm/s.

Fig. 3*A* first illustrates the primary morphological instability of the planar ice-water interface leading to the formation of cell walls. The initial breakdown of the planar front resembles the classic Mullins-Sekerka instability (19, 46), which amplifies random wavy perturbations of the interface. The following nonlinear evolution of those wavy perturbations, however, is strikingly different. Instead of forming cellular/dendritic array structures, the initially rounded protuberances evolve highly anisotropically into an array of flat ice lamellae aligned perpendicularly to the c-axis, with the corresponding templated structure shown in Fig. 3*B*. As they develop, ice lamellae interact via the solute diffusion field and grow competitively. As a result, leading lamellae grow faster while lagging ones become eliminated. so that the spacing between surviving lamellae in the right-most frame of Fig. 3*A* (primary spacing λ ) is much larger than the initial wavelength of instability of the planar interface in the left-most frame of Fig. 3*A*.

Key to explaining the formation of unilateral features is the fact that the solid-liquid interface has a completely different morphology on the two sides of the ice lamellae, which are slightly tilted with respect to the thermal axis due to the finite misorientation, γ_0_. As seen on the last frame of Fig. 3*A*, on the side pointing towards the warm side, which is exactly perpendicular to the c-axis, the interface is faceted leading to the formation of smooth featureless cell walls in the templated structure. In contrast, on the opposite side pointing towards the cold side, the interface is atomically rough, and hence subject to additional (secondary and higher-order) morphological instabilities that shape the various unilateral features observed in the templated structures including “ridges”, wrinkled cell walls, “shark-teeth”, and “jellyfish-caps” and “tentacles.”

**Fig. 4.**
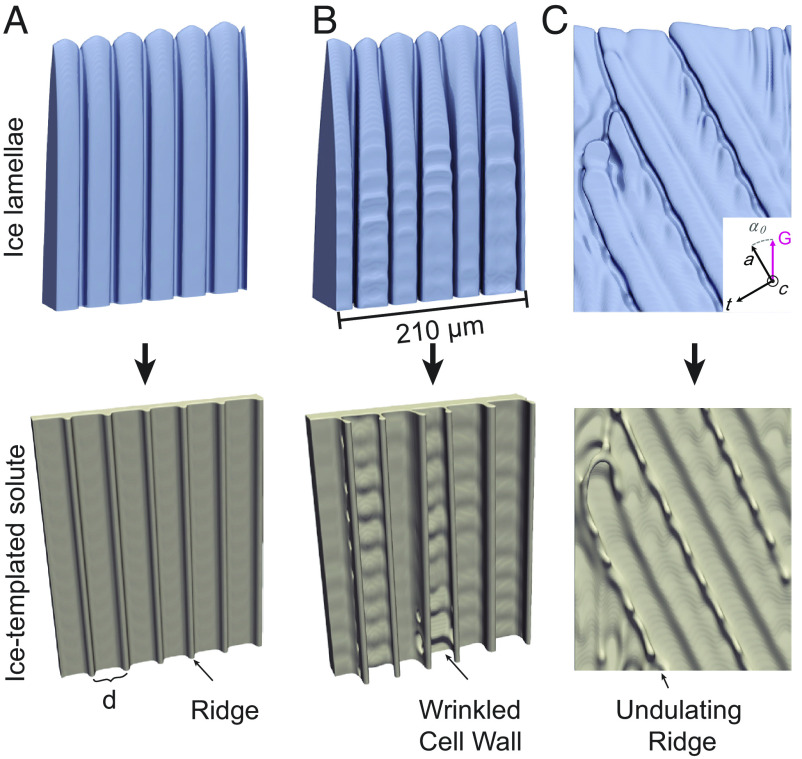
Formation mechanisms of “ridges”, wrinkled cell walls, and undulating “ridges”. The *Top* row shows the ice-crystal, and the *Bottom* row shows the corresponding ice-templated solute, where the length along the t-axis is 210 μm; the lamellar spacing λ in (*A*) and (*B*) are 50 μm and 80 μm, respectively. The wrinkled cell wall is templated in (*B*) due to a large λ . (*C*) “Shark-teeth”-like, undulating “ridges” templated by ice-crystal growth with an angle $\alpha_{0}$ = 30° between the a-axis and the temperature gradient *G* within a plane that contains both a-axis and t-axis. Growth conditions for freezing an aqueous 3% w/v trehalose solution are *G* = 12 K/cm and *V* = 15 μm/s in all simulations.

To highlight first the instability that forms “ridges”, we show in Fig. 3*C* the time evolution of an ice lamella that was created by extending to 3D, along one of the perpendicular $\langle 1\bar{1}00\rangle$ direction, a 2D steady-state growth solution (*SI Appendix*, Fig. S5). By construction, this ice lamella is initially structureless on both sides (left-most frame of Fig. 3*C*). As time evolves, however, the leading edge of the growing ice lamella is seen to become morphologically unstable on the rough side due to the destabilizing effect of the solutal diffusion field. This secondary instability occurs by a qualitatively similar mechanism to the primary Mullins-Sekerka instability of the planar interface, but it occurs along the leading edge of the growing ice lamellae. This secondary instability gives rise to the formation of a cellular structure on the rough side of the lamellae with a cell spacing that is smaller than the primary spacing, λ . The liquid in the channels in between the cells on the rough side is increasingly enriched in solute (Fig. 3*D*) and templated by the ice phase to form regularly spaced unilateral “ridges” and other features until the solute vitrifies, as illustrated in Fig. 4*A* for a similar case as in Fig. 3*D*. Interestingly, the formation of these unilateral “ridges” on the cell walls requires no other factors such as mechanical forces or fluid flow in between ice-crystals. While this “ridge”-forming instability was purposely illustrated in Fig. 3*C* for a special initial condition (structureless lamellae), the same instability occurs concurrently with the development of ice lamellae in the full 3D evolution of Fig. 3*A*.

## Wrinkled Cell Walls, “Shark-Teeth,” and “Jellyfish”

**Fig. 5.**
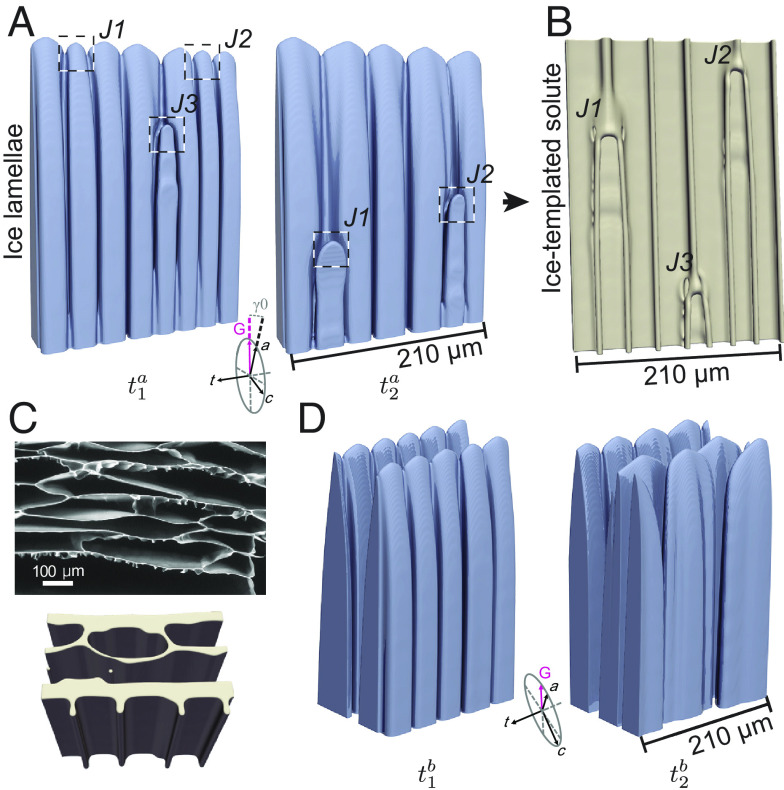
Formation mechanisms of additional microstructures due to the ice-lamellae growth. (*A*) Ice lamella at two-time steps in a PF-simulation, where three ice tips *J*_*1*_, *J_2_*, and *J_3_* are eliminated within the lamella. (*B*) The “jellyfish”-like substructures on the scaffold caused by each tip elimination event. (*C*) The upper image shows porous microstructures of the ice-templated solute observed in the experiment. The lower image shows similar porous microstructures in the PF-simulation. (*D*) Ice-crystals at two timesteps in a PF-simulation, where a regular ice lamella can evolve and become irregular. Growth conditions for freezing an aqueous 3% w/v trehalose solution are *G* = 12 K/cm and *V* = 15 μm/s in all PF-simulations.

Figs. 4 *B* and *C* and 5 *A* and *B* reveal how the additional unilateral features that decorate the cell walls result from tertiary morphological instabilities, i.e., instabilities occurring on the rough sides of ice lamellae after their formation by the primary instability of the planar interface (Fig. 3*A*) and the further breakdown of the rough side into a cellular structure by the secondary instability (Fig. 3*B*). Metallurgical cellular/dendritic structures are known to be stable for a wide range of primary spacing (38, 57, 58). Similarly here, lamellar structures with “ridges” are stable for a large range of λ . If λ is too small, lamellae are eliminated by the growth competition with other lamellae, leading to an increase of the average spacing. While, if λ is too large, new lamellae can branch out of existing lamellae to reduce the average spacing. Simulations reveal that, when λ is close to this upper limit of stability, cells on the rough side of ice lamellae develop side undulations in the direction perpendicular to the lamellae (*Top* of Fig. 4*B*). Those undulations then form wrinkled cell walls on the corresponding templated structures (*Bottom* of Fig. 4*B* corresponding to feature #5 of Fig. 2). In contrast, for smaller λ , undulations are absent and only smooth cell walls with “ridges” are formed (Figs. 3 *C* and *D* and 4*A*). Independently of the formation of unilateral features, the large stability range of primary spacing revealed by the simulations (*SI Appendix*, Table S1) also explains, why highly aligned, continuous porosity can extend through the height of the entire sample (~75 to 100 mm), despite the local cooling rate gradually changing with distance of the solidification front from the bottom of the mold (*SI Appendix*, Fig. S7).

Fig. 4*C* further reveals that “shark-teeth” form due to lateral undulations of cells on the rough sides of ice lamellae, which form when the a*-*axis contained within the basal plane of ice is misoriented by an angle $\alpha_{0}$ with respect to the thermal axis. When $\alpha_{0}=0$ (Figs. 3 *A*–*D* and 4 *A* and *B*), the cells forming on the rough side do not branch laterally. In contrast, when $\alpha_{0}$ is finite, cells drift transversely along the direction of $\alpha_{0}$ and develop sidebranches that grow towards the warm side. Those sidebranches generate undulations of the cross-sectional area of the tubular solute-rich liquid grooves, which then template the undulating “ridges” referred to as “shark-teeth”, corresponding to feature #2 of Fig. 2. While the value of $\alpha_{0}$ is hard to determine precisely from the experimental templated structures, the fact that both straight and undulating “ridges” are reasonably aligned with the thermal axis suggests that $\alpha_{0}$ is typically small. Interestingly, when $\alpha_{0}=30^{\circ }$ , as in the simulation example of Fig. 4*C*, two of the $\langle 11\bar{2}0\rangle$ preferred growth directions are symmetric about the thermal axis. Consequently, undulated “ridges” can in principle form that are tilted $\pm 30^{\circ }$ with respect to the thermal axis in the plane of ice lamellae, as observed in simulations with spatially extended lamellae. This case is analogous to the case where two equivalent $\langle 100\rangle$ dendrite growth directions make a $\pm 45^{\circ }$ angle with respect to the thermal axis during directional solidification of binary alloys with an underlying cubic symmetry (59, 60). However, a major difference here is that the smaller $\pm 30^{\circ }$ angles produce tilted dendritic structures confined to the rough side of ice lamellae (Fig. 4*C*), as opposed to degenerate “seaweed” structures (59, 60).

Fig. 5 *A* and *B* reveal the mechanism of formation of “jellyfish” features. When the solute is locally significantly upconcentrated at the freezing front, and the secondary ridge spacing falls below a threshold value, one or more competitively growing secondary ice cells can become trapped by a “jellyfish”-like polymer cap (feature #3 in Fig. 2).

The cap forms between two ice-templated polymer “ridges” and becomes eliminated by their neighbors accompanied by an adjustment of the ridge spacing. Qualitatively similar instabilities are observed in directional solidification of binary alloys with non-faceted cellular interfaces where the cell elimination leads to an adjustment of the cellular spacing (57, 61). Meanwhile, when neighboring cells decorating the rough side are close to each other, upconcentrated solute can be trapped inside enclosed tubular liquid channels. Those channels then template the “jellyfish-tentacles” that can be partially or fully detached from the ridge-free cell wall behind it (feature #4 in Fig. 2). This feature is usually observed on the solute cell wall at a sample height below the “jellyfish-caps,” where the local ridge spacing is small.

## Pore Morphologies

On a larger scale than the unilateral features that decorate the cell walls, which we have discussed so far, the cell walls themselves form a honeycomb-like pore structure illustrated in Figs. 3*B* and 5*C*. The short pore dimension, *S* = λ -*w*, where *w* is the cell wall thickness, is approximately equal to λ in the present dilute mixtures that yield very thin walls ( w≪S ). The ratio of the long and short pore dimensions varies in the range of 3 to 5, with this ratio remaining approximately constant as λ varies with processing parameters as discussed further below. The PF-simulations have enabled us to distinguish two different mechanisms that prevent lamellae from extending infinitely along the $\langle 1\bar{1}00\rangle$ directions, thereby forming the observed anisotropic honeycomb-like porosity.

The first mechanism illustrated in Fig. 3 *A* and *B* involves the randomness associated with both the initial breakdown of the planar interface that amplifies small infinitesimal random perturbations of the interface, and the transient growth competition between forming ice lamellae that eliminates lagging lamellae to coarsen the short pore dimension. Both factors contribute to forming lamellae with finite and varying lateral dimensions. Most lamellae are sufficiently wide to form a cellular structure on the rough side (with solute enriched liquid grooves between cells templating “ridges”), and typically extend a few cells, while the narrowest lamellae can comprise only a single cell. The second mechanism, illustrated in Fig. 5*D* with the corresponding templated structure at the bottom of Fig. 5*C*, involves an instability of the growing ice lamellae.

The simulation of Fig. 5*D* is, by design, free of random perturbations. It uses as an initial condition for a 3D PF-simulation an array of smooth and regular ice lamellae created by laterally extending the steady-state solution of a 2D PF-simulation. The secondary instabilities on the rough side of smooth ice lamellae give rise to a cellular morphology with additional substructures formed on the same side. Meanwhile, step-like defects form on the faceted side of ice lamellae and eventually split the lamellae to create elongated pores as illustrated in *SI Appendix*, Fig. S6. Initially, at *t* = 120 s, the step-like defect is found near the solidification front where the rough interface of a cellular structure connects with the faceted interface through sharp cellular tips. The defect is initially formed just below a groove in between cell tips. As ice lamellae drift transversely, the defect propagates down the faceted side and eventually splits the initially smooth facet. This splitting can in turn cause secondary cells to detach from an ice lamella (Fig. 5*C* and *SI Appendix*, Fig. S6), resulting in a morphological transition (of the scaffold) from regular lamellae to a porous structure similar to the experimental observation in Fig. 5*D*.

We expect both of the above “intra-grain mechanisms” to contribute to the formation of the anisotropic irregular honeycomb-like pore structures with the first and second being more dominant during the earlier and later stages of growth, respectively. On an even larger scale of the whole sample, the simultaneous growth of grains of different crystallographic orientations is reflected in the presence of larger domains of different average lamellar orientation (Fig. 2*A*). The growth competition between different grains, which is not considered here, provides an additional inter-grain mechanism to limit the size of lamellae.

## Processing-Structure Relationships: Lamellar and Ridge Spacings

**Fig. 6.**
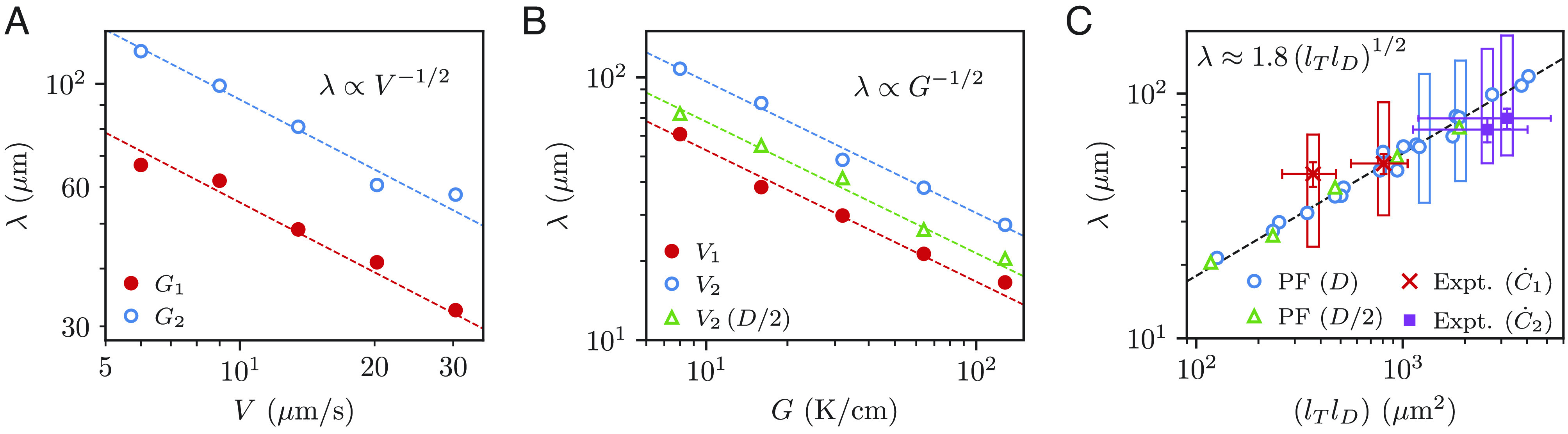
Selection of the primary spacing λ . (*A*) Circles represent 2D PF-simulations at two temperature gradients *G*_1_ = 21.9 K/cm and *G*_2_ = 9.4 K/cm. (*B*) Simulations at two velocities *V*_1_ = 28.43 μm/s and *V*_2_ = 7.62 μm/s. Green triangles represent simulations with a reduced diffusivity D/2 at *V*_2_, where *D* = 140.7 μm^2^/s. (*C*) Experimental measurements of λ at applied cooling rates of ${\dot{C}}_{1}$ = 10 °C/min and ${\dot{C}}_{2}$ = 1 °C/min are compared with the collapsed simulation results in (*A*) and (*B*), where rectangular boxes represent stable spacing ranges identified by PF-simulations (*SI Appendix*, Table S1). The dashed lines are fitted simulation results. The cross symbols with error bars are experimental values ( λ : mean ± SD; $l_{T}l_{D}$ : mean ± SE from the estimation of *G* and *V*). The dashed lines are fitted simulation results. Both simulation and experiment were performed for the freezing of an aqueous 3% w/v sucrose solution. *G*_1_ and *V*_1_ correspond to conditions at a sample height of 10mm measured from the mold bottom at an applied cooling rate ${\dot{C}}_{1}$ ; *G*_2_ and *V*_2_ correspond to a sample height of 30.5 mm freeze cast at ${\dot{C}}_{2}$.

The dominant length scale characterizing ice-templated structures is the lamellar spacing λ=S+w , where S is the short pore dimension and w is the lamellar width. Since the present experiments use dilute mixtures, w≪S and therefore λ≈S . This length scale is directly analogous to the primary spacing of dendrite array structures formed by directional solidification of metallurgical alloys, which is known to depend on the growth rate V and temperature gradient G through a scaling law of the form $\lambda ∼V^{-a}G^{-b}$ , where a and b are exponents with the empirically determined values a≈1/4 and b≈1/2 (58, 59, 62–65). In order to characterize the dependence of λ on V and G , we carried out an extensive series of 2D PF-simulations in which V was varied at two different constant values of G , and G was varied at two different constant values of V . The initial condition in those simulations consisted of a planar interface at the liquidus temperature. The average dynamically selected values of λ are obtained from multiple series of 2D PF-simulations. Snapshots of a PF-simulation at two timesteps are shown in *SI Appendix*, Fig. S5 with the accompanying movie (Movie S2). Even though the simulations are 2D, we performed a few 3D simulations to check that the 2D and 3D dynamically selected spacings are nearly indistinguishable. This is not surprising because the lamellar spacing is established by a lamellar elimination process (Fig. 3*A*) that is similar in 2D and 3D. The results shown in Fig. 6 *A* and *B*, respectively yield $\lambda ∼V^{-1/2}$ (Fig. 6*A*) and $\lambda ∼G^{-1/2}$ (Fig. 6*B*).

In order to combine those two scaling laws, we make use of the known property that directional solidification of binary alloys is controlled by three independent length scales (46, 51): the thermal length $l_{T}=\;\left|m\right|c_{\infty }/G$ that is the characteristic scale of variation of concentration on the liquid side of the interface with position along the temperature gradient, where m and $c_{\infty }$ are the liquidus slope and the sample composition, respectively, the diffusion length $l_{D}=D/V$ , which is the characteristic width of the solutal boundary layer ahead of the growing ice lamellae, and the microscopic capillary length scale $d_{0}=\Gamma /\left(\left|m\right|c_{\infty }\right)$ . Dimensional analysis suggests the combined scaling law

[2] $$\lambda =A{\left(l_{T}l_{D}\right)}^{1/2},$$

where A is a dimensionless prefactor of order unity.

While dimensional analysis alone does not exclude a dependence of this prefactor on dimensionless ratios of $d_{0}$ and $l_{D}$ or $l_{T}$ , our simulation results show that A remains approximately constant ( A≈1.8 ) for the range of growth conditions investigated (Fig. 6*C*). Importantly, this scaling law is obeyed when simulations are repeated with a diffusivity that is half of its measured value (results labeled D/2 in Fig. 6 *B* and *C*), confirming that λ is determined by the interplay of solute diffusion and the temperature gradient, as predicted by Eq. **2**. The capillary length $d_{0}$ is expected to control the radius of curvature ρ of the atomically rough region of the growing tips of ice lamellae, but this morphological feature was not investigated in detail here to test the expected scaling $\rho \;\sim \;{\left(d_{0}l_{D}\right)}^{1/2}$ (17, 26).

Dendrite array structures are also known in a metallurgical context to exist for a wide range of spacing comprised in a range $\lambda_{min}<\lambda <\lambda_{max}$ , where the ratio $\lambda_{max}/\lambda_{min}$ of the maximum ( $\lambda_{max})$ and minimum spacing ( $\lambda_{min})$ depends generally on growth conditions (27–29, 38, 57, 66–70). We carried out a separate simulations to characterize this range using the same procedure as in refs. 38, 61, and 71. which consists of performing a series of simulations with a single lamella and periodic boundary conditions perpendicularly to the thermal axis, where λ is increased or decreased in small steps. For $\lambda <\lambda_{min}$ lamellar elimination occurs leading to an increase of spacing, while for $\lambda >\lambda_{max}$ a new lamella is formed by branching from the atomically rough side of the lamella leading to a decrease of spacing. In between those two extremes ( $\lambda_{min}<\lambda <\lambda_{max}$ ) the lamellar array is morphologically stable. The stable ranges of spacing are reported in Fig. 6*C*, which indicate that, like the dynamically selected spacing governed by Eq. **2**, both $\lambda_{min}$ and $\lambda_{max}$ are proportional to ${\left(l_{T}l_{D}\right)}^{1/2}$.

In order to compare our simulation predictions with experiments in 3% w/v water-sucrose solutions, we need to take into account the fact that the crystal growth front velocity V and thermal gradient $G={\dot{C}}_{\mathrm{local}}/V$ , where ${\dot{C}}_{\mathrm{local}}$ is the local cooling rate, vary with the cooling rate $\dot{C}$ applied to the mold bottom, and the mold height H . Using a thermocouple (TC) mold (42), we determined *V* and G at regular intervals along the mold height (*SI Appendix*, Fig. S7 and Table S1). The variation of ${\dot{C}}_{\mathrm{local}}$ with H is plotted in *SI Appendix*, Fig. S7 for the two global cooling rates ${\dot{C}}_{1}$ =10 °C/min and ${\dot{C}}_{2}$ =1 °C/min, showing a steeper variation of ${\dot{C}}_{\mathrm{local}}$ vs. H for the larger global cooling rate.

To compare experiments and PF-simulations, we superimpose in Fig. 6*C* measured values of λ obtained by X-ray microtomography at different H with the corresponding values of $l_{T}l_{D}=\;\left|m\right|c_{\infty }D/\left(GV\right)$ computed using the independently measured values of V and G at the same H . Remarkably, the measured λ values for the two cooling rates fall within the range of stable spacing $\lambda_{min}<\lambda <\lambda_{max}$ . However, the dynamically selected λ values in the simulations differ from measured values. This difference is not surprising since it is well-established both experimentally (29, 72–75) and theoretically (27, 38, 57) that the dynamically selected primary spacing of dendritic arrays depends on the history of the evolution of the solid-liquid interface, which is different here in simulations and experiments.

The simulations start from a monocrystalline planar interface at the liquidus temperature and are carried out at constant isotherm V and G . In contrast, the experiments involve nucleation and then growth of ice-crystals of different orientations, so that the solidification front is initially polycrystalline and not simply planar before lamellae form. In addition, values of V and G slowly decrease with increasing mold height. However, the comparison between experiments and simulations remains meaningful since grains (regions of the same crystal orientation) that survive the initial growth competition extend laterally over several lamellar spacings. Measured spacings are therefore theoretically expected to fall within the predicted stable range of spacing, as seen in Fig. 6*C*.

Even though we have, in the present study, primarily focused on characterizing the lamellar spacing, we also measured the spacing, *d*, and height, *h*, of the “ridges”, which both increase with a decrease in applied cooling rate. In PF-simulations for 3% w/v sucrose, *d* varies from 42.1 ± 7.0 μm to 32.6 ± 2.5 μm with *V* increasing from 10 to 20 μm/s at a constant *G* = 12 K/cm. This result is in good agreement with the experimentally determined values for ridge spacing in 3% w/v sucrose scaffolds of *d =* 51.8 ± 9.2 μm at a sample position where *V* = 7.62 ± 0.12 μm/s and *G* = 9.4 ± 5.9 K/cm (*SI Appendix*, Fig. S3 and Table S1). In the case of chitosan (1.7% w/v in 1.0625% v/v acetic acid in water), the values increase from *d* = 25 ± 4 μm and *h* = 7 ± 3 μm at 10 °C/min to *d* = 43 ± 7 μm and *h* = 21 ± 2 μm at 1 °C/min, respectively (3, 4).

The PF-model can quantitatively describe also some smaller scale features in the binary water-sugar system and the ternary chitosan-acetic-acid-water system. We report here a few illustrative examples. For 3% w/v sucrose, we obtained a wavelength of 39.0 ± 6.5 μm at a ridge spacing of *d* = 77 ± 5 μm (Fig. 4*C*) for the “shark-teeth”-like undulating “ridges”, marked as feature (2) in Fig. 1, and a wavelength of 26.3 ± 2.4 μm at a ridge spacing of *d* = 35 ± 5 μm (Fig. 4*B*) for the “wrinkle”-like undulations, marked as feature (5) in Fig. 1). In the case of the ternary 1.7% w/v chitosan system, we obtained a wavelength of 73 ± 14 μm for the “shark-teeth”-like undulating “ridges” (*SI Appendix*, Fig. S8*A*) with a ridge spacing of *d* = 97 ± 4 μm, and a wavelength of 31 ± 11 μm for the “wrinkle”-like undulations (*SI Appendix*, Fig. S8*B*) with a ridge spacing of *d* = 52 ± 5 μm. For comparison, experimental measurements in the ternary chitosan-acetic-acid-water system yield a wavelength of 13 ± 8 μm at a ridge spacing of *d* = 13 ± 2 μm for “shark-teeth”-like features, and a wavelength of 26 ± 8 μm at a ridge spacing of *d* = 34 ± 7 μm for “wrinkle”-like undulations (determined on Fig. 2*F*, assuming a lamellar incline of 30° in the image). The experimentally determined wavelengths and ridge spacings tend to be smaller than those predicted by PF-simulations in the ternary chitosan-acetic-acid-water system, but still have comparable magnitudes. Since the measured and predicted ridge spacings are in good quantitative agreement in the binary water-sugar system, this quantitative discrepancy in the ternary system is likely due to the fact that this system was modeled here for simplicity as a pseudo-binary system with only acetic-acid as the mobile solute.

## Discussion

Complex, multicomponent slurries composed of large polymers or particles and additives have, to date, been too difficult to model quantitatively beyond linear stability analysis (21–23), which can only predict the initial breakdown of the planar interface. As a result, 3D ice-templated structures have not been reproduced by any model, and the mechanism of their formation has remained elusive. At the technical level, an important difficulty is that particles interact with the freezing front in multiple ways, which depend on the liquid carrier viscosity, particle material properties such as density, thermal conductivity, and particle size, size distribution, shape, surface charges and particle-particle interactions, which also determine whether particles are pushed ahead or engulfed by the freezing front; systems that contain particles are therefore difficult to model (76). An additional and possibly the main challenge is posed by the fact that the diffusion coefficient of many polymers and particles is highly nonlinear and depends on concentration and particle volume fraction, increasing by orders of magnitude as the volume fraction approaches its upper bound corresponding to the close packing limit (21).

In the present study, ice-templated structures were systematically studied and quantified in experiments performed with binary water-sugar solutions with fast diffusing solutes, which obey simple Fickian diffusion. We find that those structures exhibit strikingly similar 3D morphological features as structures formed in more complex solutions of a large polymer and additive studied here for comparison (Fig. 2). This finding is important for two reasons. First, in and of itself, it already suggests that those features may result from the strongly anisotropic properties of ice, which is the main property distinguishing binary water mixtures with small solutes from directional solidification of binary metallic alloys**.** Second, it has enabled us to validate this hypothesis by carrying out fully quantitative 3D PF-simulations of ice-templating, which reproduce for the first time all the observed ubiquitous structures and explain their formation.

To date, the formation of unilateral features has been interpreted as a manifestation of dendritic sidebranching (e.g., figure 3.10 of ref. 16) by analogy with dendritic array structures formed in directional solidification of metallic and transparent alloys with weakly anisotropic and atomically rough solid-liquid interfaces (17, 26). This analogy, however, falls short of explaining, even qualitatively, the fully 3D unilateral features (1 to 5) shown in Fig. 1. In particular, unlike secondary dendrite branches that generally grow perpendicularly to the thermal axis in crystals with cubic symmetry, “ridges” are aligned parallel to the growth direction of the solidification front and have no direct analog in metallurgical binary alloy solidification.

Our 3D PF-simulations explain how those structures form (Figs. 3–5) as a direct consequence of the strongly anisotropic properties of ice-crystal growth. While the interplay of the anisotropies of interface excess free-energy and kinetics are well-known to influence crystal growth shapes, the shapes generally reflect, on a macroscopic scale, the underlying symmetry of the crystal in most well-studied examples, e.g., snowflakes exhibit a great variety of shapes, but all have hexagonal symmetry in the basal plane (35), metallurgical dendrites in crystals with cubic symmetry typically grow in <100> directions (17, 26, 33), and surface growth shapes are typically interpreted in terms of equilibrium shapes and kinetic Wulff plots (77–80). Exceptions, such as understanding anomalous dendrite growth directions comprised between <100> and <110> directions (30, 81), have required non-trivial explanations invoking competing anisotropies of the solid-liquid interface free-energy that favor different directions.

Here, our simulations reveal how different anisotropic properties of the ice-liquid interface shape 3D unilateral features that do not reflect on a macroscopic scale the underlying symmetry of ice. They show that the two-orders of magnitude slower growth along the c-axis, compared to basal directions (strong kinetic anisotropy), is responsible for the formation of a large featureless facet on one side of the inclined lamellae. Second, the weak (of the order of one percent) interface free-energy anisotropy in the basal plane enables a regular cellular array to form on the rough side. This array templates the rectilinear “ridges” (feature 1 in Fig. 1*D*) observed experimentally, and secondary instabilities of those unilateral cellular fronts can form more complex features, such as cellular elimination forming “jellyfish”-like structures (Fig. 5). To further illustrate the role of this weak anisotropy, we have carried out a 3D simulation in which the interface free-energy is completely isotropic while the kinetic anisotropy is left unchanged. The result (*SI Appendix*, Fig. S9) shows that cellular growth on the rough side becomes unstable, generating a disordered templated structure instead of the regular “ridges” shown in Fig. 4*A*.

Finally, PF-modeling has enabled the prediction of a stable range of lamellar spacing, λ, and thereby the short pore axis, *S*, of scaffolds for binary solutions for which the fundamental physical parameters are known. The obtained scaling law of Eq. **2** offers an alternative to the, to date, empirically obtained structure-processing correlations, based on time-consuming experimentation. Already in simple binary solutions, PF-modeling is highly non-trivial because of the strongly anisotropic properties of ice-crystal growth that were incorporated quantitatively here for the first time. While, as an extension of the present work, it may be feasible to model complex slurries in the future, the newly gained understanding of how complex morphological features form in our comparatively simple binary solutions, is a critical first step. The results close a knowledge gap in the fundamental science of ice-crystal growth.

## Conclusions and Outlook

The present study has revealed for the first time how anisotropic ice-crystal growth forms hierarchical ice-templated structures exhibiting ubiquitous unilateral features such as aligned “ridges” and other complex morphological features, such as “jellyfish”-like caps and “tentacles,” which do not reflect in a simple way the underlying crystal symmetry of ice. Slow growth along the c-axis, together with a small misorientation of the basal plane with respect to the thermal axis, form ice lamellae that are only faceted on one side, while the weak sixfold anisotropy of the interface free-energy in the basal plane causes a regular array of “ridges” to form as a result of diffusion-controlled cellular instabilities of the solid-liquid interface occurring on the opposite rough sides; more complex features form as a result of the evolution of this cellular structure.

No other factors are necessary. Mechanical forces and interdendritic solute flow, which result from the 9% volumetric expansion of the water phase during solidification and thermally driven mold shrinkage, and which are known to play a critical role in the self-assembly and preferential orientation of the cell wall components during freeze casting, or solutal and/or gravitational forces, which affect cell wall material self-assembly — all of which had been suggested to explain the observed phenomena — may enhance the microstructure formation, but are not their primary cause.

The ideal binary sugar-water system with the solute of small molecular size investigated here is illustrative of important phenomena. We discover that the binary sugar-water model systems with trehalose and sucrose, which contain only fast diffusing small molecules exhibit hierarchical features that are remarkably similar to those of the ternary chitosan-acetic-acid-water system, which contains two solutes, a slow diffusing large (chitosan) and a fast-diffusing small (acetic acid) polymer. To test the hypothesis that, in the ternary system, the fast-diffusing solute controls the pattern formation process, we carried out a PF-simulation treating the ternary chitosan-acetic-acid-water system as a pseudo binary system with only acetic-acid as the mobile solute (*SI Appendix*, Fig. S4). The results exhibit similar patterns as those observed in the ternary system, which supports our hypothesis. However, the question remains, whether it is the fast-diffusing solute that dominates structure formation so that it is always necessary, for example as an additive to large polymer solutions or particle slurries. Answering this important question will be possible by extending the present PF-model to treat the highly nonlinear diffusion process associated with large solutes complemented by controlled experiments. These hypotheses are important extensions yet to be explored.

In concert, the reported mechanisms, experimental and simulation results are expected to provide a basis for the custom-design and manufacture of a wide range of free-cast materials for applications ranging from those in biomedicine to energy generation and storage.

## Materials and Methods

### Materials and Solution Preparation.

Chitosan (95% degree of deacetylation, 150 to 300 kDa; Heppe Medical Chitosan GmbH, Germany), acetic acid (Glacial, ACS grade, EMD Millipore, Burlington, MA, USA), sucrose, and trehalose (Cascade Biochem Ltd., Reading, UK) were used as received. Sucrose and trehalose solutions of 3,5 and 10% w/v in distilled water were prepared for characterizations by scanning electron microscopy (SEM) and x-ray microtomography. Sucrose and trehalose solution in deuterium oxide (D_2_O, Cambridge Isotope Laboratories, Cambridge, MA) of the 1.7, 3, 5% w/v were prepared for diffusivity measurements by Diffusion Ordered Spectroscopy (DOSY). Chitosan flakes (8 g) were soaked in deionized water for 24 h, before adding 5% v/v acetic acid (0.265 M) and raising the volume to 100 mL to prepare an 8% w/v chitosan solution. For preparation of the diluted chitosan solutions of 1.2, 1.7, 2.4, 3.6 and 4.8% w/v, the 8% w/v chitosan solution was diluted down with DI water. Similarly, 1.2, 1.7, 2.4, 3.6% w/v chitosan were dissolved in deuterium oxide with acetic acid concentrations of 0.75, 1.0625, 1.5, and 2.25% v/v, respectively, for diffusivity measurements. All solutions were homogenized in a Speed Mixer (FlackTek, Landrum, SC, USA) at 2,000 rpm for 2 min before use.

### Freeze Casting of Scaffolds.

Scaffolds were freeze cast from 1.7% w/v chitosan, and 3, 5 and 10% w/v trehalose and sucrose solutions each, using a system detailed in Wegst et al. (2). Briefly, a polytetrafluoroethylene tube (25.4 mm outer, 20 mm inner diameter, 500 mm height) was sealed with a copper mold bottom and filled with 12.5 mL of the solution using a syringe, corresponding to a filled height of 39.8 mm. The mold was then placed with its copper bottom on a liquid nitrogen cooled copper cold finger whose temperature is proportional–integral–derivative controlled. The molds were equilibrated to 4 °C for 10 min before a cooling rate of either 10 °C/min or 1 °C/min was applied until the mold reached a temperature of −150 °C. The frozen slurries were then demolded with an Arbor press and lyophilized (FreeZone 6 Plus, Labconco, Kansas City, MO, USA) for 72 h at 0.008 mbar and a coil temperature of −85 °C.

### Structural Characterization by SEM.

For structural characterization by SEM, longitudinal (parallel to the freezing direction) and transverse (perpendicular to the freezing direction) cross sections of the chitosan, sucrose and trehalose scaffolds were prepared with a razor blade (Astra Superior Platinum Double Edge) at a height of 28 mm measured from the scaffold bottom. SEM (Vega 3, Tescan, Brno-Kohoutovice, Czech Republic) was performed on non-neutralized chitosan scaffolds without prior application of a conductive coating, and sucrose and trehalose scaffold sections with 10 nm gold coating. For high resolution SEM micrographs, cell walls were carefully peeled from non-neutralized, uncoated chitosan scaffolds under a stereomicroscope (M205C, Leica Microsystems Inc., Buffalo Grove, IL, USA).

For sectioning using a focused ion beam (FIB), the peeled-off cell walls were mounted on a gold-plated (10 nm) cover slip that was placed on an SEM stub before sputter-coating also this assembly with a 10 nm thick gold layer. Taking advantage of a dual-beam system (Scios2, FEI, Hillsboro, Oregon, USA), first a 100 nm thick Pt layer was deposited by e-beam over an 42 µm by 12 µm area, then a 200 nm thick Pt layer was i-beam deposited over a 42 µm by 4 µm region of interest parallel to the “jellyfish” feature to include the entire “jellyfish-cap” as well as a small distance beyond its length. To expose the feature in cross-section, a trench was FIB-milled on an area of 40 µm by 5 µm, cutting towards the center of the feature using the milling parameters of 16 kV at 0.5 nA; milling progress was monitored by SEM. Finally, the FIB-milled cross-section was cleaned at 2 kV and 70 pA, again while closely monitoring the process by SEM, until the cutting plane met the centerline of the “jellyfish-cap.” SEM micrographs, using secondary electrons, were obtained directly after FIB-milling.

### Structural Characterization by X-ray Microtomography.

The 3D structure of scaffolds of two compositions, 3% w/v sucrose and 3% w/v trehalose, each freeze-cast at two applied cooling rates, 10 °C/min and 1 °C/min, was imaged by X-ray microtomography using a desktop system (Bruker Skyscan 1272, Kontich, Belgium) operating at a source voltage of 50 kV and a source current of 200 µA. The 360° scans were performed with a step size of 0.1°, the camera size was 2,942 × 3,280 pixels. Two voxel sizes were used, 1.5 µm and 5 µm, resulting in fields of view of 4.413 mm × 4.920 mm and 14.71 mm × 16.40 mm, respectively. Reconstructions and visualizations were obtained with the Nrecon and CTVox software tools (both Bruker, Kontich, Belgium), respectively. Pore size and geometry were determined at a height of 30.5 mm and 10.0 mm measured from the scaffold bottom with ImageJ (version 1.53c) (82).

### State Diagrams for Chitosan by Differential Scanning Calorimetry.

The phase transition temperatures for chitosan solutions were determined by differential scanning calorimetry (DSC 204, Netzsch, Selb, Germany). To perform the measurement, an aluminum crucible was filled with 10 ± 1 mL of 1.2, 2.4, 3.6, 4.8, and 8% w/v chitosan solution in 0.75, 1.5, 2.25, 3, and 5% v/v acetic acid in distilled water, respectively, and the combined mass of the sample and the crucible was determined with a precision balance (±0.01 mg; XP105 Delta Range, Mettler Toledo Inc., Columbus, OH, USA). To obtain the state diagram for chitosan, the samples were heated from −150 °C to 4 °C at a rate of 10 °C/min and the melting points were determined from the offsets of the peaks on the DSC curve (83).

### Diffusion Coefficients by DOSY.

The diffusion coefficients of chitosan, sucrose, and trehalose were determined in D_2_O with DOSY. Bipolar gradient DOSY measurements were performed for concentrations of 1.2, 1.7, 2.4, and 3.6% w/v of chitosan in acetic acid (0.75, 1.0625, 1.5, and 2.25% v/v, respectively) in D_2_O, 5% w/v sucrose in D_2_O, and 1.7%, 3%, 5% w/v trehalose in D_2_O, using a 500 Mhz Advance III system (Bruker, Billerica, MA, USA) retrofitted with a magnet with a 5 mm inner diameter probe (Oxford Instruments, Concord, MA, USA) controlled by Topspin 3.2 pl6 software (Bruker, Billerica, MA, USA). The temperature was decreased in 5 °C steps from 25 °C to 5 °C for the chitosan solutions, and in 5 °C steps from 20 °C to 5 °C for the sucrose and trehalose solutions.

### Freezing Front Velocity, Local Cooling Rate, and Temperature Gradient by TC Mold Measurements.

A custom-made TC mold was used to determine the freezing front velocity, local cooling rates, and temperature gradients at the liquid solid interface (42). The time points when the 0 °C, −5 °C and −10 °C isotherms reached each probe were plotted over the height of the probe to determine the freezing front velocity *V* [mm/s] from the first derivative of the linear (1 °C/min) or quadratic (−10 °C) fit to the height-time plot for TCs below the fill level. The TC 1 is neglected because of its proximity to the copper bottom. The local cooling rate was determined for each TC from the 0 °C to −2 °C temperature-time gradient. A linear fit of the local cooling rates over the probe height were performed, and the corrected local cooling rates ${\dot{C}}_{\mathrm{local}}$[K/s] at each probe height on the fit curve were calculated. The thermal gradients *G* [K/mm] were then calculated by ${\dot{C}}_{\mathrm{local}}$/*V*.

### PF-Simulations.

To model the unidirectional freezing of water-based solutions, we extend to ice templating the well-developed quantitative PF formulation of binary alloy solidification (51, 52). This formulation was developed to model the solidification of metallic alloys with weakly anisotropic non-faceted solid-liquid interfaces that grow at small growth rate in local thermodynamic equilibrium, corresponding to vanishing kinetic undercooling $V_{n}/\mu_{k}\left(n\right)$ in all directions in Eq. **1**. To model ice-crystal growth, we extend this formulation to interpolate between growth in local equilibrium in non-faceted directions n contained in the basal plane and slow faceted growth with a non-vanishing kinetic undercooling for n parallel to the c-axis. This extension makes use of a standard form of the anti-trapping current (51, 52), which preserves local equilibrium for growth in the basal plane, and a modified PF kinetics with a relaxation time that is both temperature- and orientation-dependent, which yields the desired strongly anisotropic form of $\mu_{k}\left(n\right)$ in the thin-interface limit of the PF-model. The present PF formulation distinguishes itself from previous formulations used to model freeze casting (24, 25) in that it incorporates quantitatively the strong anisotropy of $\mu_{k}\left(n\right)$ that is shown here to play a key role in microstructural pattern formation during ice templating. We implemented the PF-model for massively parallel computing on Nvidia Tesla V100 graphic processing units with the computer unified device architecture programming language. Full details of the model and simulation parameters are given in c.

## Supplementary Material

Appendix 01 (PDF)

Movie S1. Three-dimensional PF simulations of ice-crystal growth during directional solidification of a 3% w/v aqueous trehalose solution with growth conditions *G* = 12 K/cm and ***V*** = 15 μm/s, with the simulation domain size **337 × 216 × 216 μm^3^**, the misorientation angle γ_0_ = 10°, and the simulation time 180 s.

Movie S2. Two-dimensional PF simulations of ice-crystal growth during directional solidification of a 3% w/v aqueous trehalose solution with growth conditions *G* = 12 K/cm and ***V*** = 15 μm/s, with the simulation domain size **362 × 1208 μm^2^**, the misorientation angle γ_0_ = 10°, and the simulation time 200 s.

## Data Availability

All study data are included in the article, *supporting information*, and/or deposited in Zenodo (10.5281/zenodo.7929195) (84).
