# Supplementary material for: Hierarchical structure formation by crystal growth-front instabilities during ice templating
Source: Proc Natl Acad Sci U S A. 2023 May 31;120(23):e2210242120. doi: 10.1073/pnas.2210242120 (PMC10266019; doi:10.1073/pnas.2210242120)
Supplement: Supplementary file 1 — Appendix 01 (PDF) [file pnas.2210242120.sapp.pdf]

PNAS

Supplementary Information for

Hierarchical Structure Formation by Crystal Growth-Front Instabilities During Ice Templating

Kaiyang Yin[a,b,c,1] , Kaihua Ji[b,d,1] , Louise Strutzenberg Littles[e],
Rohit Trivedi[f], Alain Karma[b,d,*], Ulrike G.K. Wegst[a,b,*]

a Thayer School of Engineering, Dartmouth College, Hanover, NH 03755, USA
b Department of Physics, Northeastern University, Boston, MA 02115, USA
c Department of Microsystems Engineering, University of Freiburg, 79110 Freiburg, Germany
d Center for Interdisciplinary Research on Interdisciplinary Research on Complex Systems, Northeastern University, Boston, MA 02115, USA
e Materials Science and Metallurgy Branch, NASA Marshall Space Flight Center; Huntsville, AL 35812, USA
f Department of Materials Science and Engineering, Iowa State University; Ames, IA 50011, USA

[1] K.Y. and K.J. contributed equally to this work.
* Alain Karma, Ulrike G.K. Wegst
Emails: a.karma@northeastern.edu (A.K.); u.wegst@northeastern.edu (U.G.K.W.)

This PDF file includes:

Supplementary text
Figures S1 to S8
Table S1
Legends for Movies S1 and S2
SI References

Other supplementary materials for this manuscript include the following:

Movies S1 and S2

State Diagram for Chitosan by Differential Scanning Calorimetry

The phase transition temperatures for chitosan solutions were determined by differential scanning calorimetry (DSC 204, Netzsch, Selb, Germany). To perform the measurement, an aluminum crucible was filled with 10 ± 1 mL of 1.2, 2.4, 3.6, 4.8, and 8% w/v chitosan solution in 0.75, 1.5, 2.25, 3, and 5% v/v acetic acid in distilled water, respectively, and the combined mass of the sample and the crucible was determined with a precision balance (± 0.01 mg; XP105 Delta Range, Mettler Toledo Inc., Columbus, OH, USA). To obtain the state diagram for chitosan, the samples were heated from -150 °C to 4 °C at a rate of 10 °C/min and the melting points were determined from the offsets of the peaks on the DSC curve (1).

Phase-field Simulations

We extend to ice templating the well-developed quantitative phase-field (PF) formulation of binary alloy solidification (2, 3). This formulation was developed to model the solidification of metallic alloys with weakly anisotropic non-faceted solid-liquid interfaces that grow at small growth rate in local thermodynamic equilibrium, corresponding to vanishing kinetic undercooling, $\frac{V_n}{\mu_k(\boldsymbol{n})}$, in all directions in Eq. (1). To model ice growth, we extend this formulation to interpolate between growth in local equilibrium in non-faceted directions, $\boldsymbol{n}$, contained in the basal plane and slow faceted growth with a non-vanishing kinetic undercooling for $\boldsymbol{n}$ parallel to the c-axis. This extension makes use of a standard form of the anti-trapping current (2, 3), which preserves local equilibrium for growth in the basal plane, and a modified PF kinetics with a relaxation time that is both temperature- and orientation-dependent, which yields the desired strongly anisotropic form of $\mu_k(\boldsymbol{n})$ in the thin-interface limit of the PF model. The present PF formulation distinguishes itself from previous formulations used to model freeze casting (4, 5) in that it incorporates quantitatively the strong anisotropy of $\mu_k(\boldsymbol{n})$ that is shown, here, to play a key role in microstructural pattern formation during ice templating.

A phase-field, ϕ, introduced to distinguish between solid ($\phi = +1$) and liquid ($\phi = -1$) phases, is coupled to the solute concentration, c (in mole fraction). The evolution equations of ϕ and c are given by

$$\tilde{\tau}(T,\boldsymbol{n})a_s(\boldsymbol{n})^2\frac{\partial\phi}{\partial t} = \vec{\nabla}\cdot\left[a_s(\boldsymbol{n})^2\vec{\nabla}\phi\right] + \sum_i\left[\partial_i\left(\left|\vec{\nabla}\phi\right|^2 a_s(\boldsymbol{n})\frac{\partial a_s(\boldsymbol{n})}{\partial(\partial_i\phi)}\right)\right] + \phi - \phi^3 - \tilde{\lambda}(1-\phi^2)^2\frac{1}{1-k}\left[\frac{2\tilde{c}}{1+k-(1-k)\phi} - \tilde{c}_l^0(T)\right], \tag{1}$$

$$\frac{\partial\tilde{c}}{\partial t} = \tilde{D}\vec{\nabla}\cdot\left[\tilde{q}(\phi)\tilde{c}\vec{\nabla}\ln\frac{\tilde{c}}{1+k-(1-k)\phi}\right] - \vec{\nabla}\cdot\vec{j}_{at}\,, \tag{2}$$

where the index, i, sums over the spatial coordinates, i.e., $i = x, y$ in 2D and $i = x, y, z$ in 3D. The time and length are scaled by a constant relaxation time, τ_0, and the diffuse interface thickness, W, respectively; $\tilde{c} \equiv c/c_\infty$ is the dimensionless solute concentration scaled by the global sample concentration, c_∞. In Eq. (1), the scaled dimensionless relaxation time

$$\tilde{\tau}(T,\boldsymbol{n}) \equiv \tau(T,\boldsymbol{n})/\tau_0 = \tilde{c}_l^0(T) + A(\boldsymbol{n}), \tag{3}$$

is a function of both temperature and orientation, where $\tilde{c}_l^0(T) \equiv c_l^0(T)/c_\infty$ denotes the dimensionless liquidus concentration at a temperature, T, and $A(\boldsymbol{n})$ is an anisotropy function for the interface kinetics that will be discussed later in this section. The other anisotropy function, $a_s(\boldsymbol{n}) \equiv \gamma(\boldsymbol{n})/\gamma_0$, represents the excess free energy of the solid-liquid interface $\gamma(\boldsymbol{n})$ scaled by γ_0, the reference value of $\gamma(\boldsymbol{n})$ in the $\langle 1\bar{1}00\rangle$ prism direction. The coupling factor, $\tilde{\lambda} = a_1 W/d_0$, and the liquid diffusivity, $\tilde{D} \equiv D\tau_0/W^2 = a_1 a_2 W/d_0$, are two dimensionless coefficients, where $a_1 = 5\sqrt{2}/8$, $a_2 = 47/75$, the capillary length is $d_0 = \Gamma/\Delta T_0$, the Gibbs-Thomson coefficient of the solid-liquid

interface is $\Gamma = \gamma T_M/L$, and the freezing range is $\Delta T_0 = |m|c_\infty$, with T_M the melting temperature of the pure ice, L the latent heat of fusion per unit volume, and m the liquidus slope, and $k = c_s/c_l$ is the partition coefficient, with c_s and c_l the solute concentrations at the solid and liquid sides of the interface, respectively. In Eq. (2), $\tilde{q}(\phi)$ is a dimensionless function for the standard one-sided model of alloy solidification that interpolates between 0 in the solid and 1 in the liquid; $\tilde{q}(\phi)$ is a dimensionless function that interpolates between 0 in the solid and 1 in the liquid

$$\tilde{q}(\phi) = \frac{1-\phi}{1+k-(1-k)\phi} \,. \tag{4}$$

The anti-trapping current $\vec{j}_{at}$ takes the form:

$$\vec{j}_{at} = \frac{1}{\sqrt{2}}\left[\tilde{c}\partial_t \ln(1 + k - (1-k)\phi)\frac{\vec{\nabla}\phi}{|\vec{\nabla}\phi|}\right] . \tag{5}$$

The PF model presented in this paper has been adapted to simulate the ice-templating process. Unlike the solidification of binary alloys, almost all impurities are rejected by a growing ice phase during freezing at relatively small velocities (6, 7). We need to choose a reference temperature, T_0, in order to simulate the ice-templating system with a small k (the value $k = 10^{-4}$ is used in all PF simulations). In this work, T_0 is chosen such that the equilibrium concentration on the liquid side of the interface at T_0 equals to the global sample composition c_∞, i.e., $c_l^0(T_0) = (T_M - T_0)/|m| = c_\infty$. Then, we can obtain $T_0 = (T_M - |m|c_\infty)$. In the PF simulation, we consider the frozen temperature approximation that defines the temperature field directed along the vertical *x*-axis, $T(x) = T_0 + G(x - Vt)$, where G is the temperature gradient, and V is the pulling speed. The frozen temperature approximation enters the PF equations through $\tilde{c}_l^0(T)$ in Eq. (1).

For a linear liquidus, $\tilde{c}_l^0(T)$ has an explicit form

$$\tilde{c}_l^0(T) = 1 - \frac{x-Vt}{l_T} \,, \tag{6}$$

where $l_T = |m|c_\infty/G$ is the thermal length.

The linear liquidus slope can be quantitatively predicted by the Clausius-Clapeyron relation for dilute alloys (8) with $k \to 0$,

$$|m_v| = \frac{k_B T_m^2}{L}\frac{1}{m_B} \,, \tag{7}$$

where m_v denotes the liquidus slope in the unit of Kelvin per weight by unit volume, k_B is the Boltzmann constant, and m_B is the molecular mass of solute. We also derive an expression of the liquidus slope in the unit of Kelvin per weight percent. Ignoring the volume change due to mixing, we can re-express Eq. (7) in the form

$$|m| = \frac{k_B T_m^2}{L}\left(\frac{\rho_A \rho_B}{(1-c_w)m_B\rho_B + c_w m_B \rho_A}\right), \tag{8}$$

where m denotes the liquidus slope in the unit of Kelvin per weight percent, ρ_A is the density of the solvent, ρ_B is the density of the solute, and c_w is the weight fraction of the solute. In the dilute limit $c_w \ll 1$, we can obtain $|m| \approx k_B T_m^2 \rho_A / L m_B$. With water as the solvent, ρ_A = 1 g/cm^3, and we can obtain $|m| \times 1\%\ \mathrm{w/w} = |m_v| \times 1\%\ \mathrm{w/v}$. For sucrose and trehalose of the same molar mass 342.3 g/mol, the liquidus slope of their aqueous solutions in the dilute limit is

$|m_v| \approx 0.0543$ K/(% w/v) (Fig. S1A). For the chitosan-acetic-acid-water solution, the molar mass of the small solute molecule, acetic-acid, is 60.052 g/mol, and we can obtain the liquidus slope of this solution in the dilute limit $|m_v| \approx 0.31$ K/(% w/v) (Fig. S1B). Importantly, the dilute alloy approximation that assumes a constant liquidus slope includes a sufficiently large region of the solidification front to capture the key morphological instabilities of the solid-liquid interface that shape the complex microstructures.

In addition, we incorporate anisotropic energetic and kinetic properties of the ice-water interface that are consistent with the hexagonal symmetry of ice and properties derived from both experimental measurements and molecular dynamics simulations (9). The ice-water interface is faceted in the basal plane normal to the $\langle 0001 \rangle$ directions (c-axis) and typically atomically rough in other directions contained within the basal plane, including the six $\langle 11\bar{2}0 \rangle$ preferred growth directions (a-axis) and the six prism $\langle 1\bar{1}00 \rangle$ directions (t-axis). Accordingly, the excess free energy of the solid-liquid interface is chosen to have a six-fold symmetry within the basal plane perpendicular to the c-axis and a two-fold symmetry within any plane parallel to the c-axis:

$$a_s(\boldsymbol{n}) = a_s^0 f(\theta, \varphi) = a_s^0[1 + \epsilon_1 |\sin\theta| + \epsilon_6 \sin^6\theta \cos 6\varphi] \,, \tag{9}$$

where θ is the polar angle with respect to the c-axis in the plane that contains both a- and c-axes, and φ is the azimuthal angle with respect to the a-axis in the plane that contains both a- and t-axes. These two angles can be evaluated locally by the phase-field ϕ, with the c-axis parallel to the *z*-direction and the a-axis parallel to the *x*-direction in three dimensions:

$$\theta = \sin^{-1}\left(\frac{\sqrt{\phi_x^2+\phi_y^2}}{\sqrt{\phi_x^2+\phi_y^2+\phi_z^2}}\right), \tag{10}$$

$$\varphi = \cos^{-1}\left(\frac{-\phi_x}{\sqrt{\phi_x^2+\phi_y^2}}\right), \tag{11}$$

where ϕ_i denotes the partial derivative $\partial\phi/\partial i$ along the $i = x, y,$ or z direction. The second term inside the bracket on the right-hand-side of Eq. (9) corresponds to cusps in the $\langle 0001 \rangle$ directions perpendicular to the ice facets. In numerical simulations, cusps are handled by slightly rounding the anisotropy function over a narrow range of orientation (i.e., $|\sin\theta|$ is regularized by $\sqrt{\sin^2\theta + \delta^2}$ with $\delta = 0.01$). This method has been shown to converge well quantitatively to the physical sharp-cusp limit for the growth of faceted interfaces in the context of crystal growth from the melt (10) and nanowire vapor-liquid-solid growth (11). The third term inside the bracket is a spherical harmonic with hexagonal symmetry. We choose ϵ_1 and ϵ_6 values according to the ice-water interfacial free-energy estimated by molecular dynamics simulations (9). The average values taken from the literature are 25.9 $\mathrm{mJ/m^2}$ for the basal plane and 28.0 and 29.1 $\mathrm{mJ/m^2}$ for the interfaces perpendicular to the $\langle 1\bar{1}00 \rangle$ and $\langle 11\bar{2}0 \rangle$ directions, respectively. Those values yield $\epsilon_1 = 0.1$, $\epsilon_6 = 0.02$ and $a_s^0 = 0.925$, with the reference value $a_s = 1$ in the prism directions. In two dimensions, the anisotropy function has the same form, except the azimuthal angle $\varphi = 0°$ is a constant.

The interface kinetics is sufficiently fast in atomically rough directions, and the interface can be assumed in local thermodynamic equilibrium. In contrast, the growth of facets is a much slower process involving layer-by-layer growth, typically controlled by two-dimensional nucleation or spiral growth around screw dislocations (12). This interface kinetics can be described by the relationship between interface velocity, V_n, and kinetic undercooling, ΔT_k. Rewrite Eq. (1) from the main text, and we can obtain the interface undercooling ΔT measured with respect to the liquidus temperature, i.e., $\Delta T = T_M - |m|c_l - T_I$, where T_I is the interface temperature. Here ΔT contains

contributions from both the capillary and kinetic undercoolings, i.e., $\Delta T = \Delta T_c + \Delta T_k$, where $\Delta T_c = \frac{T_M}{L}\sum_{i=1}^{2}\left[\gamma(\boldsymbol{n}) + \gamma_{\theta_i\theta_i}(\boldsymbol{n})\right]\mathcal{K}_i$ is the capillary undercooling and $\mathcal{K}_i$ are the principle interface curvatures. Even though the relationship between V_n and ΔT_k obtained from experimental measurements of the basal plane growth (13–15) is generally nonlinear over a very large range of undercooling, reflecting complex mechanisms of layer-by-layer growth, it can be reasonably well approximated by a linear relationship

$$V_n = \mu_k^{\langle 0001\rangle} \Delta T_k \tag{12}$$

for the limited range of undercooling (around 0.04 K) that spans the narrow tip region of the growing partially faceted ice crystals controlling hierarchical structure formation, where $\mu_k^{\langle 0001\rangle}$ denotes the kinetic coefficient along the $\langle 0001\rangle$ facetted-growth direction. Accordingly, we incorporate the anisotropic interface kinetics into the PF model by choosing a form of the kinetic coefficient $\mu_k(\boldsymbol{n})$ that interpolates between a vanishingly small kinetic undercooling for the growth of atomically rough interfaces in directions contained within the basal plane, corresponding to a diverging value of the kinetic coefficient, and a finite value of $\mu_k^{\langle 0001\rangle}$ in directions perpendicular to the basal plane. We use the value of $\mu_k^{\langle 0001\rangle} = 41.1\ \mu\mathrm{m\,s^{-1}\,K^{-1}}$ obtained by a linear approximation of the experimentally measured velocity-undercooling relationship over the range of undercooling of interest. The thin-interface limit of the PF model (16), written down here for the brevity of notation for an isotropic interfacial free-energy in 2D, yields the velocity-dependent form of the Gibbs-Thomson relation for solute concentration

$$c_l/c_\infty = c_l^0(T)/c_\infty - d_0\mathcal{K} - \beta(\boldsymbol{n})V_n\ , \tag{13}$$

where $\beta(\boldsymbol{n}) = 1/[\mu_k(\boldsymbol{n})\,\Delta T_0]$ is an orientation-dependent coefficient. According to the asymptotic analysis (16), $\beta(\boldsymbol{n})$ can be approximated by

$$\beta(\boldsymbol{n}) \approx \frac{a_1\tau(T,\boldsymbol{n})}{\tilde{\lambda}W} - \frac{a_1 a_2 W}{D}\tilde{c}_l^0(T)\ . \tag{14}$$

Note that the weak anisotropy of interface excess free energy is not taken into account in Eq. (14). In previously modeled binary alloy systems with atomically rough interfaces in all orientations, a temperature-dependent form of the relaxation time $\tau(T)$ has been traditionally used to make the coefficient β vanish (16). Here, to make $\mu_k(\boldsymbol{n})$ a finite value on the basal plane, we have chosen a form of $\tau(T,\boldsymbol{n})$ in Eq. (3) that is a function of both temperature and orientation, such that β vanishes along directions contained within the basal plane and equals a finite value along directions perpendicular to the basal plane. To ensure $1/\tau(T,\boldsymbol{n})$ has two cusps in the $\langle 0001\rangle$ directions, the anisotropy function $A(\boldsymbol{n})$ in Eq. (3) is chosen as

$$A(\boldsymbol{n}) = A(\theta) = A_0\left[\frac{1}{1 - r(1-|\sin\theta|)/(1+r)} - 1\right], \tag{15}$$

where A_0 is a scaling parameter and r is a constant that controls how rapidly the interface kinetic coefficient varies with orientation. In the direction of the c-axis ($\theta = 0°$), $A(\theta) = A_0 r$ and $\partial A(\theta)/\partial\theta = [-A_0 r(1+r)]$, which are determined by both coefficients A_0 and r. As a result, β is independent of the temperature and is only orientation-dependent, i.e.,

$$\beta(\boldsymbol{n}) \approx \frac{a_1 a_2 W}{D}A(\boldsymbol{n})\ . \tag{16}$$

Thus, the model coefficients can be linked to the physical kinetic coefficient $\mu_k^{\langle 0001\rangle}$ through the relation $\mu_k^{\langle 0001\rangle} = D/(a_1 a_2 \Delta T_0 W A_0 r)$. In PF simulations, we set $r = 32$ and calculate A_0 to match a given $\mu_k^{\langle 0001\rangle}$ value.

To simulate ice lamellae in 3D, we first obtained an array of stable-growing ice cells in 2D. The periodic boundary condition was applied in the transverse (cell drifting) directions. Then we elongated 2D solutions of ϕ and c fields along the perpendicular $\langle 1\bar{1}00 \rangle$ directions and utilized them as initial conditions of 3D simulations. The periodic boundary condition was applied in both the transverse and $\langle 1\bar{1}00 \rangle$ directions in 3D. In all PF simulations, random noise with uniform distribution was applied in the ϕ field to represent the thermal fluctuation (17). The physical parameters used in PF simulations include the diffusion coefficients $D = 140.7\ \mu\mathrm{m}^2/s$ for a 3% w/v aqueous sugar (sucrose or trehalose) solution, $D = 463.0\ \mu\mathrm{m}^2/s$ for a 1.7% w/v chitosan in 1% v/v acetic acid in water solution, and the Gibbs-Thomson coefficient $\Gamma = 1.64 \times 10^{-8}\ \mathrm{K} \cdot \mathrm{m}$ for the ice-water interface. The model equations are solved on a square lattice in 2D and a cubic lattice in 3D using the finite-difference method with grid spacing Δx and the explicit Euler method with time step Δt. The modeling parameters used in both 2D and 3D PF simulations include: $\tilde{\lambda} = 4.64$, $\widetilde{D} = 2.90$, $\Delta x/W = 0.8$, $\Delta t/\tau_0 = 0.029$, and $A_0 = 2.24$ for the sugar-water system; $\tilde{\lambda} = 14.94$, $\widetilde{D} = 9.36$, $\Delta x/W = 0.8$, $\Delta t/\tau_0 = 0.009$, and $A_0 = 2.29$ for the chitosan-acetic-acid-water system. The misorientation angles in the PF simulation are implemented through the rotation matrix methods in 2D (18) and 3D (19). In this work, we implement one misorientation angle γ_0 in 2D, and two misorientation angles α_0 and γ_0 in 3D. By definition, γ_0 is the angle between the a-axis and *G* within the plane that contains both a- and c-axes, and α_0 is the angle between the a-axis and a reference direction (the projection of *G*) within the plane that contains both a- and t-axes.

We implemented the PF model for massively parallel computing on Nvidia Tesla V100 graphic processing units (GPU) with the computer unified device architecture (CUDA) programming language.

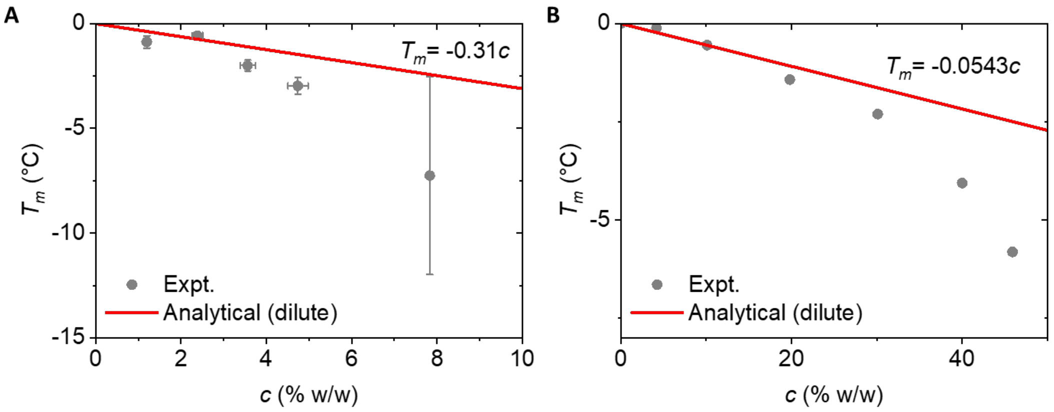

Fig. S1. (A) State diagram of sucrose-water solution in Ablett et al. (1992) (20). **(B)** State diagram plotting the melting point, T_m, of a chitosan in acetic-acid-water solution versus the chitosan concentration, c (% w/w), determined at a heating rate of 10 °C/min. Three specimens of each concentration were measured (n = 3). The analytical liquidus slopes are estimated by the Clausius-Clapeyron relation in the dilute limit (8).

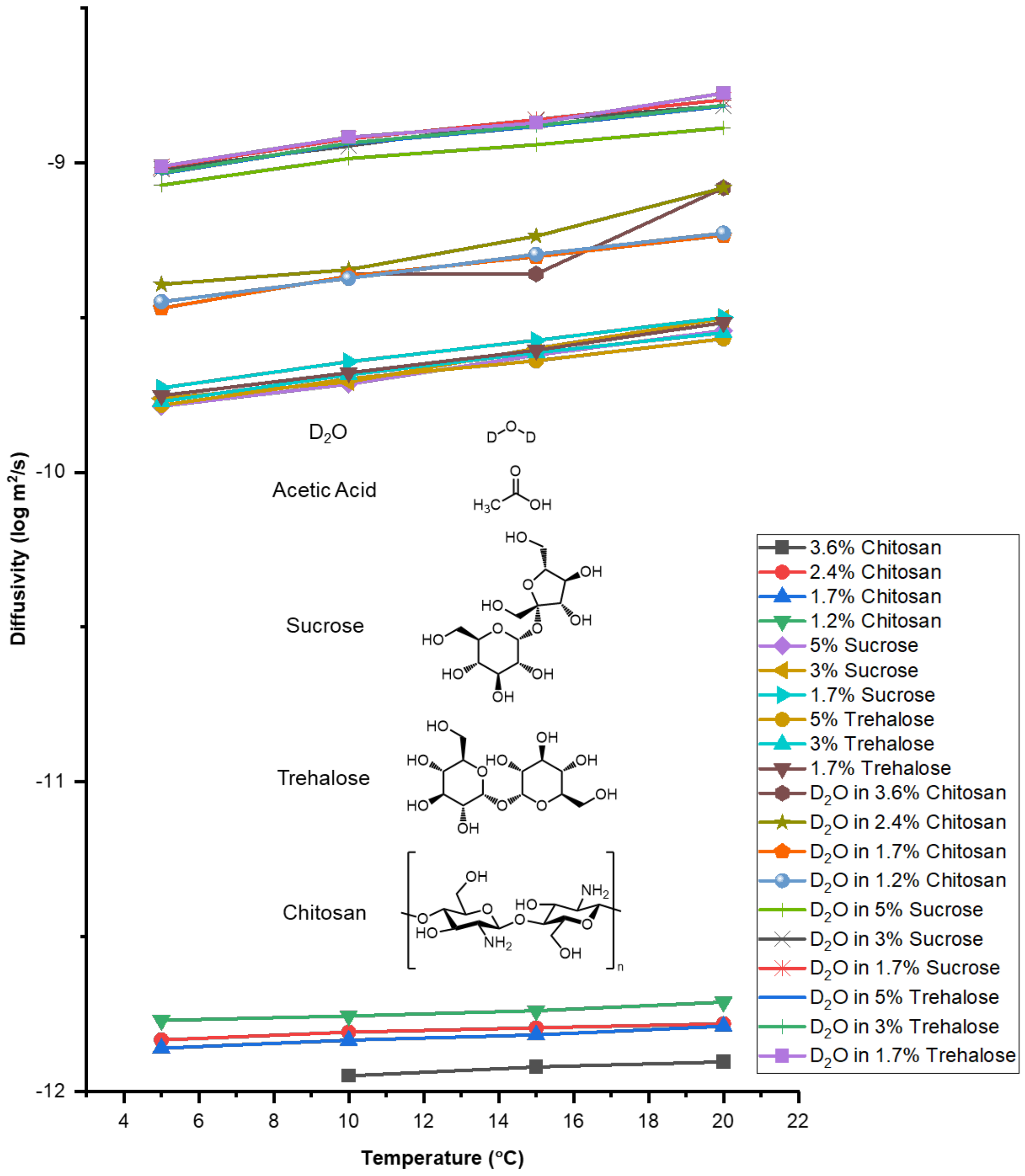

Fig. S2. Diffusivity of chitosan, acetic acid and D_2O in chitosan-acetic-acid-D_2O solution, sucrose and D_2O in sucrose-D_2O solution, and trehalose and D_2O in trehalose-D_2O solution.

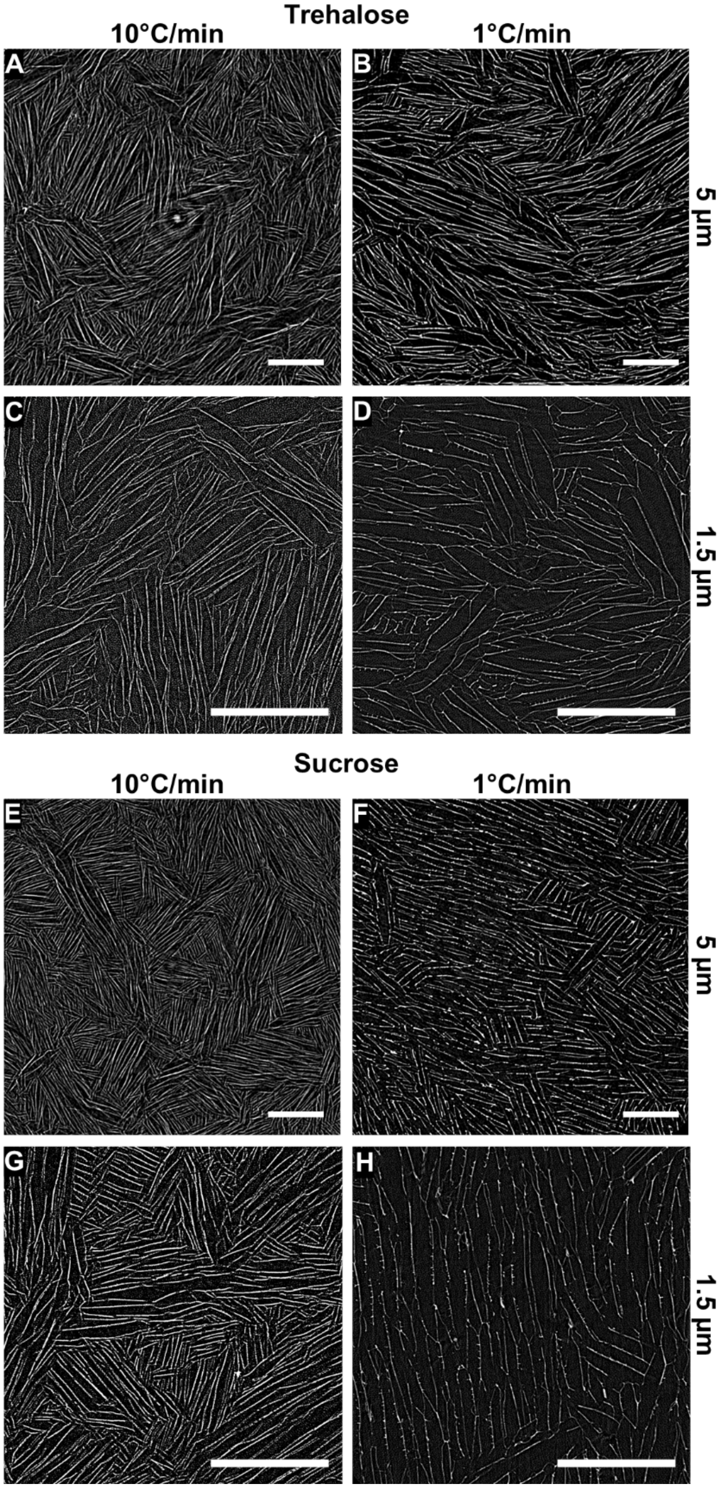

Fig. S3. A horizontal cross-sections of the reconstruction of the X-ray microtomograms of (A)-(D) 3% w/v trehalose and (E)-(H) 3% w/v sucrose scaffolds freeze cast with an applied cooling rate of (A), (C), (E), (G) 10 °C/min and (B), (D), (F), (H) 1 °C/min, imaged with a voxel resolution of (A), (B), (E), (F) 5µm and (C), (D), (G), (H) 1.5µm, respectively. The scale bars are 1mm.

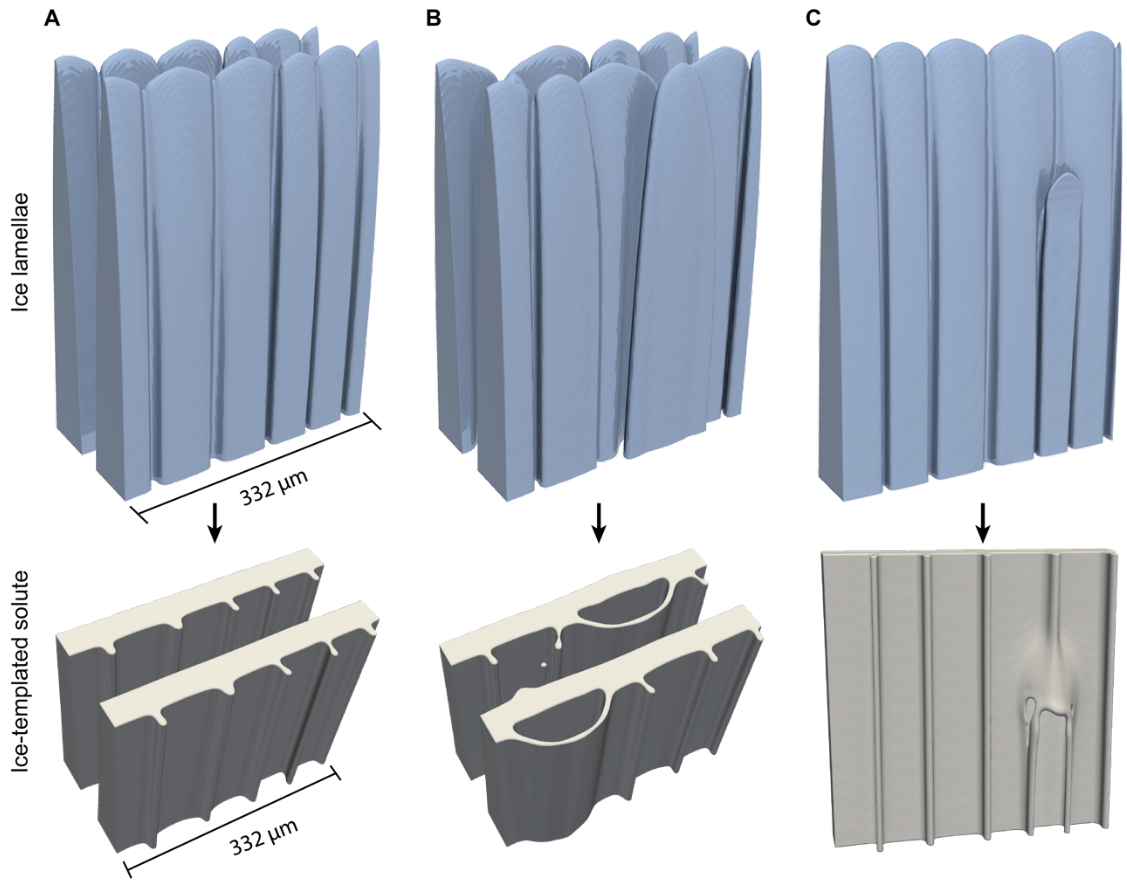

Fig. S4. Microstructural formation in PF simulations of the chitosan-acetic-acid-water system (1.7% w/v in 1% v/v acetic acid in water) with growth conditions G = 12 K/cm and $\boldsymbol{V}$ = 15 μm/s. (A) Regular ice lamellae (primary instabilities) with secondary instabilities on the cell walls that template "ridges" in the polymer phase. (B) Irregular ice lamellae that cause the formation of additional features in the templated polymer phase with porous structures. (C) The cell-tip elimination that causes the "jellyfish"-like feature in the polymer phase.

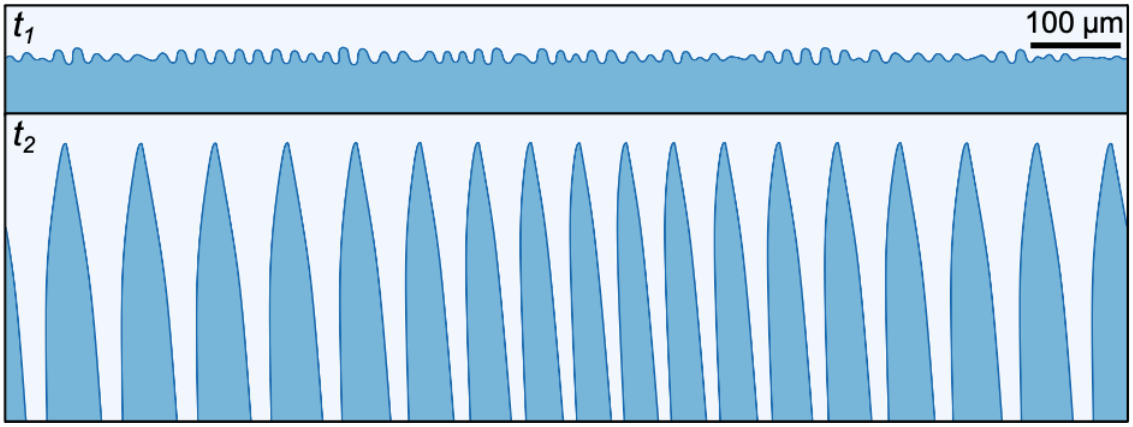

Fig. S5. Two-dimensional PF simulations of ice-crystal growth during directional solidification of a 3% w/v aqueous trehalose solution. The growth conditions are G = 12 K/cm and $\boldsymbol{V}$ = 15 μm/s. The accompanying movie for this simulation is Movie S2.

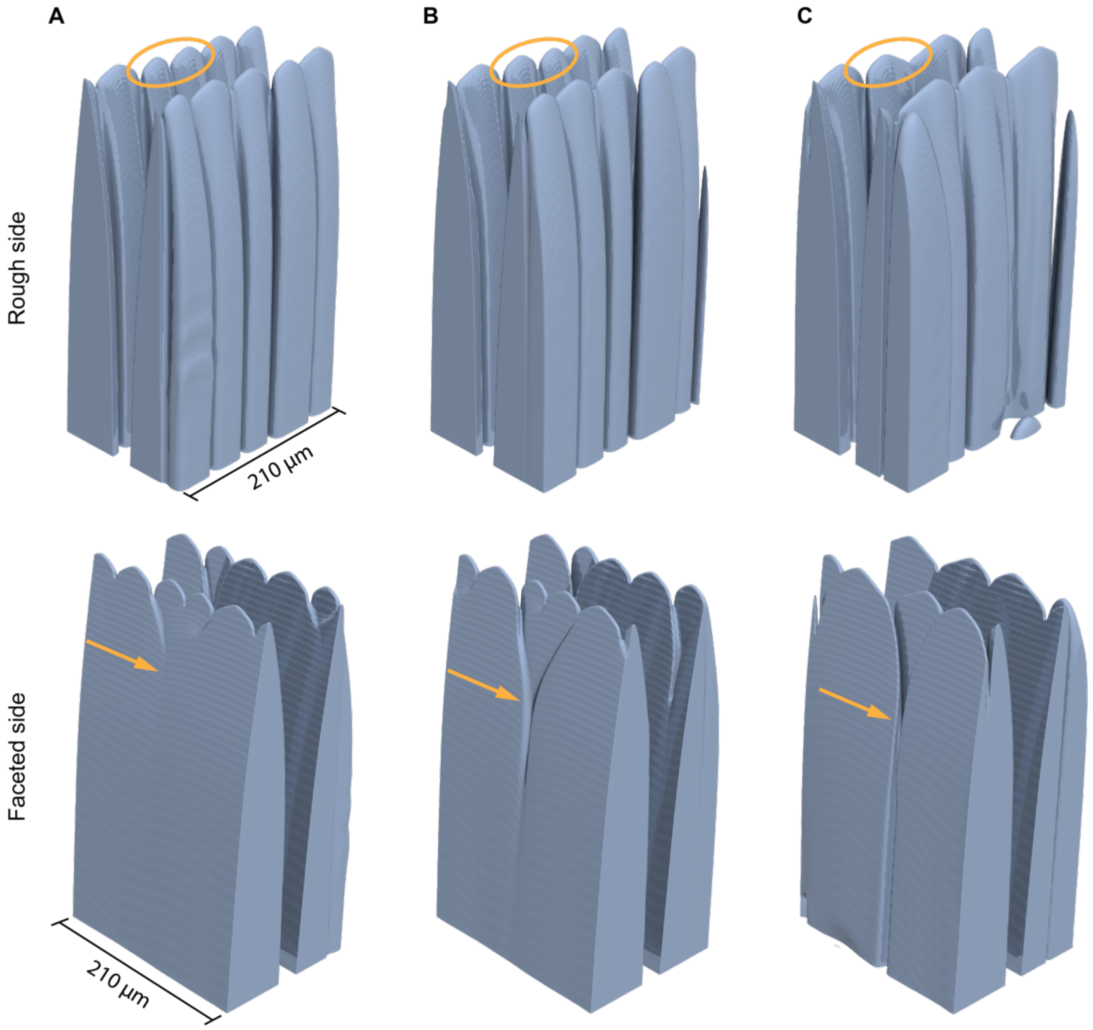

Fig. S6. Morphological instabilities on the faceted side of the ice lamellae. Ice lamellae captured at (A) t = 120 s, (B) t = 150 s, and (C) t = 210 s in the PF simulation with regular ice lamellae as the initial condition. The top and the bottom rows show the rough and the faceted sides of ice lamellae, respectively. The defect on the faceted side (marked by arrows) can develop and lead to the detachment of cells (marked by circles) from a regular lamella. The PF simulation corresponds to Fig. 5D of the main text for freezing an aqueous 3% w/v trehalose solution with G = 12 K/cm and $\boldsymbol{V}$ = 15 μm/s.

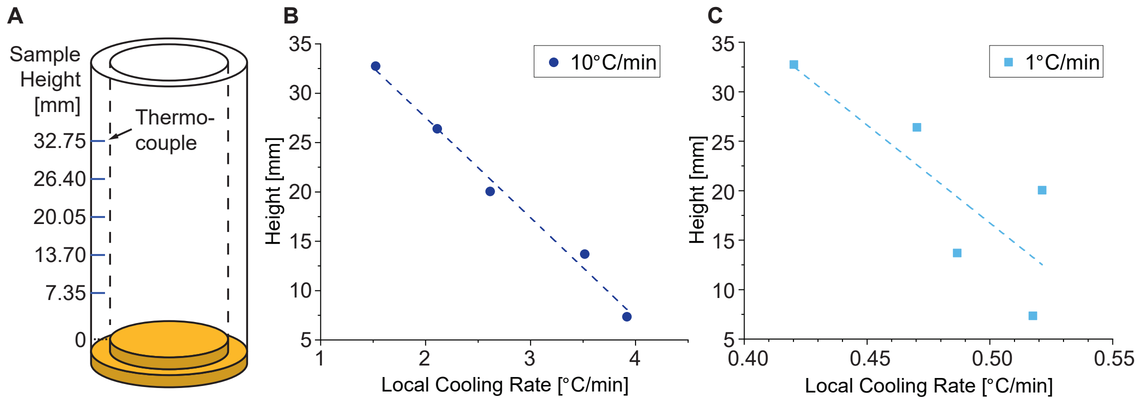

Fig. S7. Thermocouple mold (TC mold) measurements of local cooling rate along the height, *H*, of the mold. (A) Schematic of the thermocouple mold. (B) Local cooling rate $\dot{C}_{local}$ along *H* during freeze casting of 3% w/v sucrose solution at 10 °C/min applied cooling rate. (B) Local cooling rate $\dot{C}_{local}$ along *H* during the freeze casting of a 3% w/v sucrose solution at a 1 °C/min applied cooling rate.

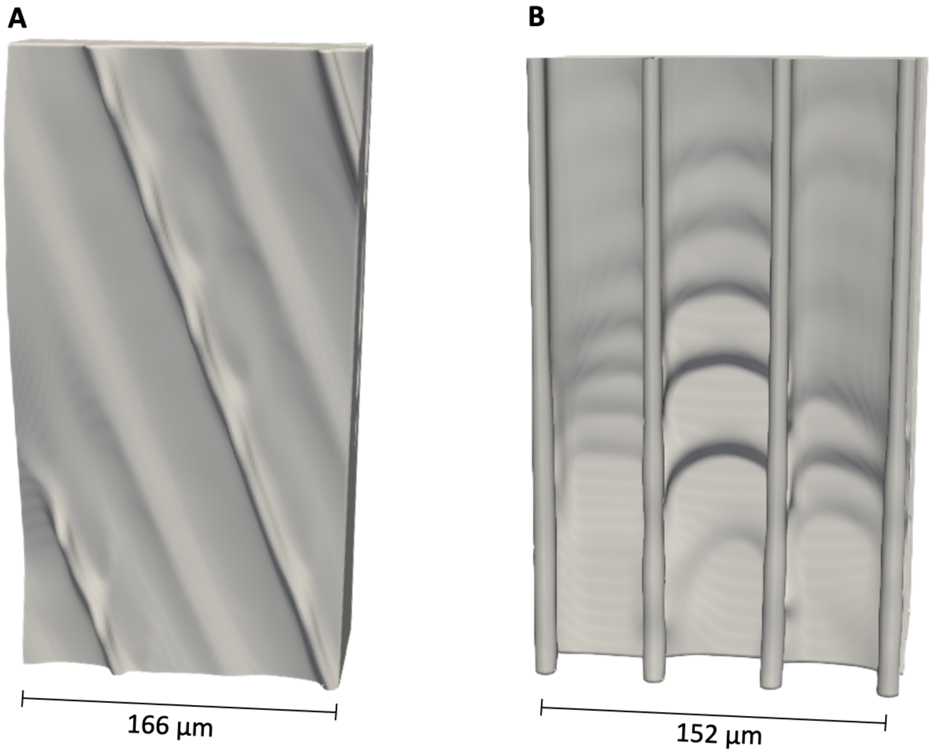

Fig. S8. (A) Wrinkled cell wall and (B) "shark teeth"-like, undulating "ridges" templated by ice-crystal growth in PF simulations of the chitosan-acetic-acid-water system (1.7% w/v in 1% v/v acetic acid in water) with growth conditions G = 12 K/cm and $\boldsymbol{V}$ = 15 µm/s.

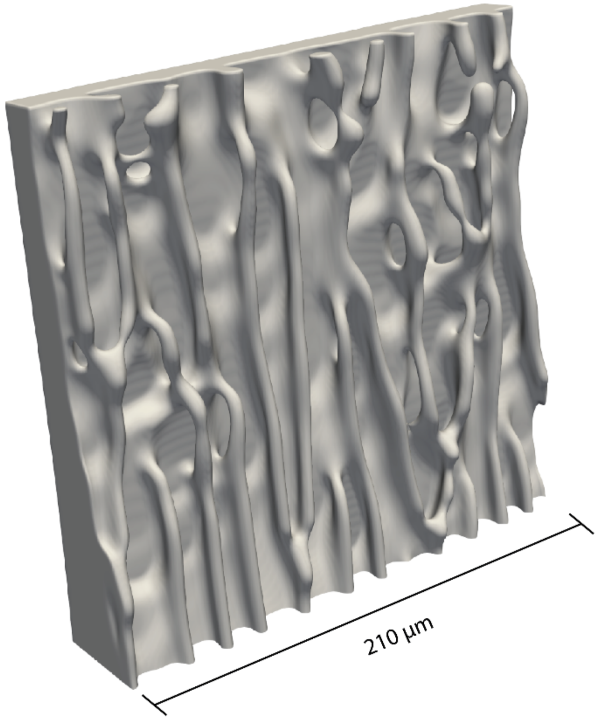

Fig. S9. Simulated templated structure obtained assuming isotropic interface free-energy anisotropy ($\boldsymbol{a_s^0 = 1}$, $\boldsymbol{\epsilon_1 = 0}$ and $\boldsymbol{\epsilon_6 = 0}$) in the basal plane of ice. The PF simulation was performed with the same growth conditions as Fig. 5A of the main text for freezing an aqueous 3% w/v trehalose solution with G = 12 K/cm and $\boldsymbol{V}$ = 15 µm/s.

Table S1. Freezing front velocity, $\boldsymbol{V}$, local cooling rate, $\dot{\boldsymbol{C}}_{\boldsymbol{local}}$, and thermal gradients, G = $\dot{\boldsymbol{C}}_{\boldsymbol{local}}/\boldsymbol{V}$, during freeze casting of trehalose and sucrose scaffolds at 10 °C/min and 1 °C/min applied cooling rates, and the lamellar spacing $\boldsymbol{\lambda}$ (mean ± SD) of the resulting scaffolds measured on X-ray microtomograms (Fig. S3). The measurements were performed at 30.5 mm and 10.0 mm mold height, *H*. The average lamellar spacing, $\boldsymbol{\lambda}$ (mean ± SD), is also measured in two-dimensional PF simulations with a domain size $\mathbf{362} \times \mathbf{804}\ \boldsymbol{\mu}\mathbf{m}^{\mathbf{2}}$. The limits of the spacing stability $\boldsymbol{\lambda}_{min}$ and $\boldsymbol{\lambda}_{max}$ are determined by progressively varying $\boldsymbol{\lambda}$ in PF simulations, and their errors equal to the varying step (4 µm). The lower limit $\boldsymbol{\lambda}_{min}$ is linked to an elimination instability, while the upper limit $\boldsymbol{\lambda}_{max}$ is linked to a tertiary branching instability.

Composition	Applied Cooling Rate	Sample Height	Freezing Front Velocity	Local Cooling Rate	Thermal Gradient	Lamellar Spacing	(PF) Lower Lamellar Spacing Limit	(PF) Upper Lamellar Spacing Limit
	$\dot{C}$	H	V	$\dot{C}_{local}$	G	λ	λ_{min}	λ_{max}
	[°C/min]	[mm]	[µm/s]	[K/min]	[K/mm]	[µm]	[µm]	[µm]
3% w/v Sucrose	10	30.5	16.59 ± 3.29	1.71 ± 0.21	1.71 ± 0.40	51.80 ± 4.87	31.8 ± 4.0	92.2 ± 4.0
		20.0	23.41 ± 4.64	2.72 ± 0.24	1.95 ± 0.42			
		10.0	28.43 ± 5.63	3.73 ± 0.29	2.19 ± 0.47	46.97 ± 5.42	23.7 ± 4.0	68.0 ± 4.0
	1	30.5	7.62 ± 0.12	0.43 ± 0.27	0.94 ± 0.59	79.19 ± 7.54	55.9 ± 4.0	172.6 ± 4.0
		20.0	7.62 ± 0.12	0.48 ± 0.29	1.06 ± 0.62			
		10.0	7.62 ± 0.12	0.53 ± 0.30	1.17 ± 0.66	71.28 ± 8.09	51.9 ± 4.0	152.5 ± 4.0
PF Modeling			10		1.2	80.4 ± 9.6	43.9 ± 4.0	136.4 ± 4.0
			15		1.2	56.2 ± 2.3	35.8 ± 4.0	120.3 ± 4.0
			20		1.2	50.5 ± 1.7	31.8 ± 4.0	112.3 ± 4.0
			15		0.8	77.8 ± 7.9	39.8 ± 4.0	148.5 ± 4.0
			15		1.2	56.2 ± 2.3	35.8 ± 4.0	120.3 ± 4.0
			15		1.6	55.6 ± 7.5	31.8 ± 4.0	104.2 ± 4.0

Movie S1 (separate file). Three-dimensional PF simulations of ice-crystal growth during directional solidification of a 3% w/v aqueous trehalose solution with growth conditions *G* = 12 K/cm and $\boldsymbol{V}$ = 15 μm/s, with the simulation domain size $\mathbf{337} \times \mathbf{216} \times \mathbf{216}\ \boldsymbol{\mu}\mathbf{m}^{\mathbf{3}}$, the misorientation angle $\boldsymbol{\gamma_0} = \mathbf{10}^{\circ}$, and the simulation time 180 s.

Movie S2 (separate file). Two-dimensional PF simulations of ice-crystal growth during directional solidification of a 3% w/v aqueous trehalose solution with growth conditions *G* = 12 K/cm and $\boldsymbol{V}$ = 15 μm/s, with the simulation domain size $\mathbf{362} \times \mathbf{1208}\ \boldsymbol{\mu}\mathbf{m}^{\mathbf{2}}$, the misorientation angle $\boldsymbol{\gamma_0} = \mathbf{10}^{\circ}$, and the simulation time 200 s.

SI References

1. ASTM International, "D3418-15 Standard test method for transition temperatures and enthalpies of fusion and crystallization of polymers by differential scanning calorimetry" (ASTM International, 2015), DOI: 10.1520/D3418-15 (25 April 2023).

2. B. Echebarria, R. Folch, A. Karma, M. Plapp, Quantitative phase-field model of alloy solidification. *Phys. Rev. E* **70**, 061604 (2004).

3. A. Karma, Phase-field formulation for quantitative modeling of alloy solidification. *Phys. Rev. Lett.* **87**, 115701 (2001).

4. M. Seiz, B. Nestler, Modelling and simulation of the freeze casting process with the phase-field method. *Comput. Mater. Sci.* **193**, 110410 (2021).

5. T.-H. Huang, *et al.*, Phase-Field Modeling of Microstructural Evolution by Freeze-Casting. *Adv. Eng. Mater.* **20**, 1700343 (2018).

6. A. W. Rempel, M. G. Worster, The interaction between a particle and an advancing solidification front. *J. Cryst. Growth* **205**, 427-440 (1999).

7. S. S. L. Peppin, M. G. Worster, J. S. Wettlaufer, Morphological instability in freezing colloidal suspensions. *Proc. R. Soc. Math. Phys. Eng. Sci.* **463**, 723–733 (2007).

8. A. Karma, Fluctuations in solidification. *Phys. Rev. E* **48**, 3441–3458 (1993).

9. R. L. Davidchack, R. Handel, J. Anwar, A. V. Brukhno, Ice Ih–Water Interfacial Free Energy of Simple Water Models with Full Electrostatic Interactions. *J. Chem. Theory Comput.* **8**, 2383–2390 (2012).

10. J.-M. Debierre, A. Karma, F. Celestini, R. Guérin, Phase-field approach for faceted solidification. *Phys. Rev. E* **68**, 041604 (2003).

11. N. Wang, M. Upmanyu, A. Karma, Phase-field model of vapor-liquid-solid nanowire growth. *Phys. Rev. Mater.* **2**, 033402 (2018).

12. D. P. Woodruff, *The Solid-Liquid Interface* (CUP Archive, 1973), pp. 151-173.

13. A. S. Michaels, P. L. T. Brian, P. R. Sperry, Impurity Effects on the Basal Plane Solidification Kinetics of Supercooled Water. *J. Appl. Phys.* **37**, 4649–4661 (1966).

14. W. B. Hillig, "The kinetics of freezing of ice in the direction perpendicular to the basal plane" in *Growth and Perfection of Crystals*, R. H. Doremus, B. W. Roberts, D. Turnbull, Eds. (Wiley, 1958), pp. 350–360.

15. K. G. Libbrecht, Physical Dynamics of Ice Crystal Growth. *Annu. Rev. Mater. Res.* **47**, 271–295 (2017).

16. B. Echebarria, R. Folch, A. Karma, M. Plapp, Quantitative phase-field model of alloy solidification. *Phys. Rev. E* **70***, 061604 - Stat. Phys. Plasmas Fluids Relat. Interdiscip. Top.* (2004).

17. B. Echebarria, A. Karma, S. Gurevich, Onset of sidebranching in directional solidification. *Phys. Rev. E* **81***, 021608 - Stat. Nonlinear Soft Matter Phys.* (2010).

18. D. Tourret, A. Karma, Growth competition of columnar dendritic grains: A phase-field study. *Acta Mater.* **82**, 64–83 (2015).

19. D. Tourret, Y. Song, A. J. Clarke, A. Karma, Grain growth competition during thin-sample directional solidification of dendritic microstructures: A phase-field study. *Acta Mater.* **122**, 220–235 (2017).

20. S. Ablett, M. J. Izzard, P. J. Lillford, Differential scanning calorimetric study of frozen sucrose and glycerol solutions. *J. Chem. Soc. Faraday Trans.* **88**, 789–794 (1992).
